# Interaction of Tau, IL-6 and mitochondria on synapse and cognition following sevoflurane anesthesia in young mice

**DOI:** 10.1016/j.bbih.2020.100133

**Published:** 2020-08-28

**Authors:** Jie Zhang, Yuanlin Dong, Lining Huang, Xiaoming Xu, Feng Liang, Sulpicio G. Soriano, Yiying Zhang, Zhongcong Xie

**Affiliations:** a Department of Anesthesiology, Tongji Hospital, Tongji Medical College, Huazhong University of Science and Technology, Wuhan, 430030, PR China; b Department of Anesthesia, Critical Care and Pain Medicine, Massachusetts General Hospital and Harvard Medical School, Charlestown, MA, 02129-2060, USA; c Department of Anesthesiology, The Second Hospital of Hebei Medical University, Shijiazhuang, PR China; d Department of Forensic Clinical Medicine, School of Forensic Medicine, China Medical University, Shenyang, PR China; e Department of Anesthesiology, Perioperative and Pain Medicine, Boston Children’s Hospital, Harvard Medical School, Boston, MA, 02115, USA

**Keywords:** Tau, IL-6, Mitochondria, Synapse, Cognition, Young mice

## Abstract

Tau phosphorylation is associated with cognitive impairment in young mice. However, the underlying mechanism and targeted interventions remain mostly unknown. We set out to determine the potential interactions of Tau, interleukin 6 (IL-6) and mitochondria following treatment of anesthetic sevoflurane and to assess their influences on synapse number and cognition in young mice. Sevoflurane (3% for 2 ​h) was given to wild-type, Tau knockout, IL-6 knockout, and cyclophilin D (CypD) knockout mice on postnatal (P) day 6, 7 and 8. We measured amounts of phosphorylated Tau, IL-6, reactive oxygen species (ROS), mitochondrial membrane potential (MMP), ATP, postsynaptic density 95 (PSD-95), synaptophysin, N-cadherin, synapse number, and cognitive function in the mice, employing Western blot, electron microscope and Morris water maze among others. Here we showed that sevoflurane increased Tau phosphorylation and caused IL-6 elevation, mitochondrial dysfunction, synaptic loss and cognitive impairment in young wild-type, but not Tau knockout, mice. In young IL-6 knockout mice, sevoflurane increased Tau phosphorylation but did not cause mitochondrial dysfunction, synaptic loss or cognitive impairment. Finally, sevoflurane increased Tau phosphorylation and IL-6 amount, but did not induce synaptic loss and cognitive impairment, in young CypD knockout mice or WT mice pretreated with idebenone, an analog of co-enzyme Q10. In conclusion, sevoflurane increased Tau phosphorylation, which caused IL-6 elevation, leading to mitochondrial dysfunction in young mice. Such interactions caused synaptic loss and cognitive impairment in the mice. Idebenone mitigated sevoflurane-induced cognitive impairment in young mice. These studies would promote more research to study Tau in young mice.

## Highlights

• Research in context.
  • Evidence before this study
    a. Tau, a microtubule-associated protein that is predominantly expressed inside neurons, is associated with microtubule assembly and function. Tau phosphorylation, aggregation and spread all serve as the pathogenesis of age-dependent neurodegeneration in the old brain, as well as the neuropathogenesis of Alzheimer’s disease. However, the effects of Tau on the cellular changes and the function of the young brain are undetermined. Our previous studies showed that anesthetic sevoflurane induced Tau phosphorylation, IL-6 elevation, mitochondrial dysfunction and synaptic loss in brain tissues of neonatal mice, as well as cognitive impairment in the mice. However, the potential interactions of the Tau phosphorylation, IL-6 elevation and mitochondrial dysfunction and the influences of these interactions on synapse number and cognitive function in neonatal mice remains largely unknown.
  • Added value of study
    b. Employing sevoflurane as a clinically relevant tool, and using the approaches including wild-type, Tau, IL-6, and CypD knockout neonatal mice, the present studies showed that Tau phosphorylation caused IL-6 elevation, which induced mitochondrial dysfunction, leading to synaptic loss and cognitive impairment in the neonatal mice. Idebenone, a synthetic analog of coenzyme Q10, mitigated the sevoflurane-induced cognitive impairment in the neonatal mice.
  • Implications of all the available evidence
    c. These findings demonstrated the role of Tau phosphorylation in cognitive impairment in neonatal mice, revealed the effects of the interactions of Tau phosphorylation, IL-6 elevation and mitochondrial dysfunction on the synapse number and cognitive function in the mice, and identified potential targeted intervention of the cognitive impairment in the neonatal mice.

## 1 Introduction

Our previous studies showed that anesthetic sevoflurane increased Tau phosphorylation in brain tissues of young mice and induced cognitive impairment in the young mice (Tao et al., 2014; Yu et al., 2020). However, the underlying mechanisms by which Tau phosphorylation is associated with cognitive impairment in young mice are mostly unknown. Moreover, the targeted intervention(s) of the cognitive impairment in the young mice remain largely to be determined.

Anesthesia has been reported to cause neuroinflammation (Shen et al., 2013), induce mitochondrial dysfunction (Amrock et al., 2015; Boscolo et al., 2012, 2013; Sanchez et al., 2011; Sun et al., 2016) and synaptic loss (Briner et al., 2011; Head et al., 2009; Lunardi et al., 2010) in the brain tissues of young rodents and monkeys. Specifically, anesthetic sevoflurane increased Tau phosphorylation (Tao et al., 2014; Yu et al., 2020) and IL-6 amounts (Shen et al., 2013; Tao et al., 2014), caused mitochondrial dysfunction (Xu et al., 2017) and induced synaptic loss (Lu et al., 2017; Tao et al., 2014; Xu et al., 2017; Zhang et al., 2015) in brain tissues of young mice.

However, whether there are interactions and dependency of Tau, IL-6 and mitochondrial function in young mice is poorly known. Specifically, whether the Tau phosphorylation, IL-6 elevation and mitochondrial dysfunction following the sevoflurane anesthesia are dependent on each other in the young mice remains unknown. We therefore employed the young mice with knockout (KO) of Tau, IL-6 and Cyclophilin D (CypD), as the tools, to study the interactions and dependency of Tau, IL-6 and mitochondria and the influences of such interactions on synapse number and cognitive function in young mice following sevoflurane anesthesia.

Postsynaptic density-95 (PSD-95) is an excitatory postsynaptic marker (Chen et al., 2015; Coley and Gao, 2018), synaptophysin is a synaptic plasticity-related protein (Hao et al., 2017; Janz et al., 1999; Xiang et al., 2018), and N-cadherin is an adhesion molecule between cells accounting for synaptic plasticity (Basu et al., 2015; Cen et al., 2018; Chazeau et al., 2015; Nagaoka et al., 2014). Reductions of these synaptic markers suggest synaptic loss (Clare et al., 2010; Hong et al., 2016; Kaufman et al., 2015; Murmu et al., 2015). Mitochondrial dysfunction includes increases in the amounts of reactive oxygen species (ROS), and reduction in the amounts of mitochondrial membrane potential (MMP) and concentrations of ATP (Brookes et al., 2004; Chaturvedi and Flint Beal, 2013; Valero, 2014). We, therefore, measured the amounts of ROS, MMP, ATP, and the synaptic markers, as well as the numbers of synapses in the hippocampi of young mice after the treatment with anesthetic sevoflurane.

CypD is the component of the mitochondrial permeability transition pore (mPTP) (Johri and Beal, 2012; Zhu et al., 2013). KO of CypD can stabilize mitochondrial function (Du et al., 2008, 2011; Fischer et al., 1989; Gainutdinov et al., 2015; Zhang et al., 2019) and mPTP is involved in anesthesia-induced mitochondrial dysfunction (Zhang et al., 2012). CypD KO mice, Tau KO mice and IL-6 KO mice were, therefore, used in the present studies to determine the interactions and dependency of Tau phosphorylation, and IL-6 accumulation in the young mice following the treatment with sevoflurane.

Idebenone is a synthetic analog of co-enzyme Q10 with the effects of protecting mitochondrial function (e.g., antioxidant and increases in ATP amounts) (Erb et al., 2012; Haefeli et al., 2011; Nagaoka et al., 1989; Voronkova and Meleshkov, 2009). We, therefore, assessed whether idebenone could mitigate the sevoflurane-induced increase in Tau phosphorylation and cognitive impairment in the young mice.

The objective of the study was to determine the underling mechanisms by which Tau phosphorylation is associated with cognitive impairment in young mice. We specifically used anesthetic sevoflurane as a clinically relevant tool to investigate (1) whether there were interactions and dependency of Tau phosphorylation, IL-6 elevation and mitochondrial dysfunction following sevoflurane anesthesia in young mice, and (2) whether such interactions could be one of the underlying mechanisms by which Tau phosphorylation is associated with synaptic loss and cognitive impairment in young mice. We tested a hypothesis that the interactions of Tau phosphorylation, IL-6 elevation and mitochondrial dysfunction following sevoflurane treatment caused synaptic loss in young mice, leading to cognitive impairment in the mice.

We measured amounts of phosphorylated Tau and IL-6 to determine the effects of anesthetic sevoflurane on Tau phosphorylation and IL-6 elevation in wild-type (WT), Tau KO, IL-6 and CypD KO young mice. We determined the concentration of ROS, levels of MMP, and concentration of ATP to assess the effects of anesthetic sevoflurane on mitochondrial function in these mice. Finally, we determined the amount of PSD-95, synaptophysin, and N-cadherin to investigate the effects of anesthetic sevoflurane on the expression of synaptic markers in these mice. We measured these changes at postnatal day (P) 8, the end of the sevoflurane anesthesia, to assess the acute effects of sevoflurane. We measured these changes at P30 because the same sevoflurane anesthesia caused cognitive impairment tested from P31 to P38 in the young mice.

## 2 Materials and methods

### 2.1 Mice anesthesia and treatment

The animal studies were conducted according to the guidelines and regulations of the National Institutes of Health (NIH). Efforts were made to minimize the number of animals used in the studies. The Standing Committee on the Use of Animals in Research and Teaching at Massachusetts General Hospital approved the studies (Protocol number: 2006N000219, Boston, Massachusetts). Since the objective of the present studies was not to determine the sex-dependent effects of sevoflurane, we did not allocate the equal number of female or male mice in each of the experimental or control groups. Rather, the mixture of female and male WT mice (C57BL/6J, Jackson Lab, Bar Harbor, ME), Tau KO mice (B6.129X1-Mapt^tm1Hnd^/J, Jackson Lab), IL-6 KO mice (B6; 129S2-Il6^tm1Kopf^/J, Jackson Lab), and CypD KO mice (B6; 129-Ppiftm1Jmol/J, Jackson Lab) were used in the studies. The mice were randomly assigned to the sevoflurane group and control group. The mice received sevoflurane from P6 to P8 and then were decapitated for the harvest of mice hippocampi at P8 or P30. We used a different group of mice in the behavioral studies. These mice received sevoflurane or control condition from P6 to P8, and then had the Morris Water Maze (MWM) test from P31 to P38. The mice in the sevoflurane group received sevoflurane (3%) plus 60% oxygen (balanced with nitrogen) 2 ​h daily for three consecutive days as performed in our previous studies (Lu et al., 2017; Shen et al., 2013; Tao et al., 2014; Xu et al., 2017; Zhang et al., 2015). The 3% sevoflurane is a clinically relevant concentration and the anesthesia with 3% sevoflurane 2 ​h daily for three days from P6 to P8 conceptually mimics the multiple exposures of anesthesia in patients. Our recent studies showed that 3% sevoflurane 2 ​h daily for 3 ​days ​at every other day (P6, P8, and P10) also caused cognitive impairment in the young mice (Lu et al., 2017). The findings that sevoflurane induced cognitive impairment in the young mice after exposure of sevoflurane at either every day or every other day suggests that the observed cognitive impairment is not due to the acute accumulation of anesthetics, instead it is due to the multiple exposures of the anesthetics. The control condition was oxygen (60% oxygen and balanced with nitrogen) with an equal rate of flow in a box which was identical to the anesthesia box (Shen et al., 2013; Tao et al., 2014). We used 60% oxygen to maintain the satisfied amounts of oxygen partial pressure, pH and carbon dioxide partial pressure in the mice after sevoflurane as performed in our previous studies (Lu et al., 2017; Shen et al., 2013; Tao et al., 2014; Xu et al., 2017; Zhang et al., 2015). Note that the mice in the control conditions were also separated from the dams and given the exposures of control gas (60% oxygen). The induction flow rate of fresh gas was 2l/minute from the start up to 3 ​min (for the purpose of induction) and then 1l/minute with the rest of the anesthesia (for maintenance). The concentrations of sevoflurane and oxygen were continuously monitored by using a gas analyzer (Dash 4000; GE Healthcare, Milwaukee, WI) during the anesthesia. The anesthesia box temperature was monitored and controlled by a feedback-based system with the DC Temperature Control System (World precision instruments, Inc Sarasota, FL, USA), which manages and automatically adjusts the temperature to keep the rectal temperature of each mouse at 37 ​°C (±0.5 ​°C) by placing a warming pad under this box. In the intervention studies, the mice were treated with idebenone (200 ​mg/kg, dissolved in DMSO and saline at the concentration of 6 μg/μl, Sigma, St. Louis, MO) (Ali et al., 2012) through intraperitoneal (IP) administration 30 ​min before each of the sevoflurane treatments on P6, P7, and P8. The mice in the vehicle group received 0.1 ​mL DMSO solution (15 ​μL DMSO dissolved in 1 ​mL saline), which was the vehicle of idebenone.

### 2.2 Harvest of brain tissues

The brain tissues of mice were harvested within 2 ​h of ending anesthesia on P8 or on P30. We did not determine the dynamic changes of Tau phosphorylation and IL-6 elevation following the sevoflurane anesthesia in the present study. Each of the mice was euthanized by decapitation at P8 or P30, and the hippocampi of the mice were harvested. The harvested hippocampi were homogenized on frost by using immunoprecipitation buffer (Tris-HCl: 10 ​mM, pH 7.4; NaCl: 150 ​mM; EDTA: 2 ​mM, Nonidet P-40: 0.5%) plus protease inhibitor cocktail from Sigma (cat# 11836170001, St. Louis, MO). Finally, the lysates were collected, which were then centrifuged 10 ​min at the speed of 12,000 ​rpm.

### 2.3 Quantification of protein

Total proteins were quantified by using a bicinchoninic acid protein assay (Pierce, Iselin, NJ) as utilized in other studies (Dong et al., 2009).

### 2.4 Western blot analysis

The quantitative Western blot was utilized in the present studies. We used antibody AT8 (55 ​kDa, 1:1000; Invitrogen, Carlsbad, CA) to detect the amounts of Tau phosphorylated at serine 202 and threonine 205 (Tau-PS202/PT205) amino acid. IL-6 antibody (24 ​kDa, 1:1000; Cat. # ab6672, Abcam, Cambridge, MA) was used to recognize IL-6. Postsynaptic density (PSD)-95 antibody (95 ​kDa, 1:1000; Cell Signaling, Danvers, MA), synaptophysin antibody (38 ​kDa, 1:1000; Cell Signaling) and N-cadherin antibody (140 ​kDa, 1:1000; Cell Signaling) were used to detect the protein amounts of PSD-95, synaptophysin, and N-cadherin, respectively. A β-Actin antibody (42 ​kDa, 1:5000; Sigma) was used to detect β-Actin. The quantification of Western blot was accomplished as described in other studies (Dong et al., 2009). In brief, we analyzed the signal intensity via Quantity One image analysis program (Bio-Rad, Hercules, CA). Two steps were used to quantify the Western blots. At the first step, β-Actin was used to standardize protein amounts (e.g., calculating the ratio of PSD-95 as compared to β-Actin amount), limiting the differences in the protein amount loaded. At the second step, we expressed the protein amounts obtained from the treatment as a percentage to the control condition.

### 2.5 ROS measurement

An OxiSelect In Vitro ROS/RNS Assay Kit (Cell Biolabs, San Diego, CA) was used to measure the amounts of ROS, according to our previous studies (Zhang et al., 2012).

### 2.6 Isolation of mitochondria

A Mitochondria Isolation Kit for Tissue (Abcam, Cambridge, MA) was used to isolate mitochondria, according to our previous studies (Zhang et al., 2012). Briefly, the harvested hippocampi were mixed with isolation buffer up to 2.0 ​mL, dounced in the pre-chilled douncing homogenizer, and then centrifuged at 1000×*g* for 10 ​min at 4 ​°C. After that, the supernatant was transferred into two new tubes with 2.0 ​mL isolation buffer in each tube and centrifuged at 12,000×*g* for 15 ​min at 4 ​°C. The pellets were collected and washed twice. Finally, the pellets were combined and resuspended in 500 ​μL isolation buffer supplemented with protease inhibitor cocktail (Sigma) for the determination of mitochondrial membrane potential (MMP).

### 2.7 Determination of MMP

We used a JC-1 Mitochondrial Membrane Potential Detection Kit (Biotium, Hayword, CA) to determine MMP amounts according to our previous studies (Zhang et al., 2012).

### 2.8 ATP measurement

We employed an ATP Colorimetric/Fluorometric Assay Kit (Biovision, Milpitas, CA) in the experiments to detect ATP amounts, according to the protocol provided by the company.

### 2.9 Electron microscope and analysis

We used the methods described in previous studies (Kovalenko et al., 2018) to determine the effects of sevoflurane on the number of synapses by using an electron microscope. We perfused each mouse with cold PBS followed by fixing solution (2% paraformaldehyde and 2% glutaraldehyde in PBS). The similar locations in the CA1 area of hippocampi were dissected out, stored at 4 ​°C overnight in the fixing solution and sliced into 1 ​mm slices on a vibratome. There were 20 brain sections collected in each mouse and each brain section had fifteen distinct apical regions. The slices were further post-fixed with 1% osmium tetroxide/PBS, dehydrated in graded ethanol (50% and 70%), and stained with 1% uranyl acetate in 70% ethanol for 1 ​h. Then, the slices were dehydrated in graded ethanol (90% and 100%), mounted in Epon resin (Marivac Canada Inc. St. Laurent, QC, Canada) on glass slides and cured overnight at 60 ​°C. Ultrathin sections (about 60 ​nm) were cut on a Reichert Ultracut-S microtome, placed onto copper grids, stained with uranyl acetate and lead citrate, and examined in a Jeol JEM 1011 transmission electron microscope. Fifteen distinct apical regions of CA1 were imaged per mouse to analyze the number of the synapses. The number of synapses was counted in a blind manner.

### 2.10 Morris water maze (MWM)

MWM experiments were performed using the methods described in our previous studies (Shen et al., 2013; Tao et al., 2014; Zhang et al., 2015). Briefly, MWM consists of a round steel pool (150 ​cm in diameter and 60 ​cm in height) that was filled with water to a height of 1.0 ​cm above the top of a 15-cm diameter platform. The pool was covered with a black curtain and was located in an isolated room with four visual cues on the wall of pool. Water was kept at 20 ​°C and opacified with titanium dioxide. We tested the mice in the MWM for seven days (P31 to P37) with four trials daily. Escape latency (the time for the mouse to reach the platform) in the MWM training from P31 to P37 was recorded to assess the mouse spatial learning function. The platform crossing numbers (the counts the mouse moved across the original area of the removed platform) in MWM probe test were recorded on P38 to assess the mouse spatial memory function. Mouse body temperature was maintained by using a heating device described in previous studies (Shen et al., 2013; Tao et al., 2014; Zhang et al., 2015).

### 2.11 Statistics

The data obtained from biochemistry studies and escape latency of MWM were presented as mean ​± ​standard deviation (SD). The numbers of the platform crossing numbers of MWM were presented as median with interquartile range (25%–75%). Two-way ANOVA with repeated measurement was used to evaluate the interaction of the difference of escape latency of the mice in the anesthesia group as compared to the mice in the control group in the MWM test. Post-hoc analysis was used to compare the change in escape latency of the mice in the anesthesia group to the mice in the control group on each day during the MWM test, and cut-off alpha was adjusted using the Bonferroni method. We used the Mann-Whitney-U test to compare the difference in the platform crossing numbers of the mice in the sevoflurane group and the mice in the control group. Two-way ANOVA without repeated measurement was used to evaluate the interaction of group and treatment on protein amounts among the WT and KO mice in the control and anesthesia group. The two-way ANOVA without repeated measurement was also used to evaluate the interaction of group and treatment on the variables among the WT mice pretreated with vehicle (DMSO) and the WT mice pretreated with idebenone. We performed these tests based on the distributional assumptions from our previous similar work. The number of animals or samples was 10–15 in each group in behavioral studies and was 9 in the biochemistry studies. The sample size was chosen empirically based on the previous studies (Lu et al., 2017; Shen et al., 2013; Tao et al., 2014; Xu et al., 2017; Zhang et al., 2015). The studies employed two-tailed hypothesis and statistically significant P values were <0.05. We used the software of Prism 8 (La Jolla, CA) to evaluate all of the data.

## 3 Results

### 3.1 Sevoflurane increased Tau phosphorylation and IL-6 elevation, induced mitochondrial dysfunction, synaptic loss and cognitive impairment in WT young mice

The objective of the present study was to use both genetic and pharmacological approaches to determine the potential interactions and dependency of Tau phosphorylation, IL-6 elevation and mitochondrial dysfunction following the treatment of sevoflurane in young mice, and to assess whether such interactions could change the synapse number and cognitive function in the mice.

**Fig. 1:**
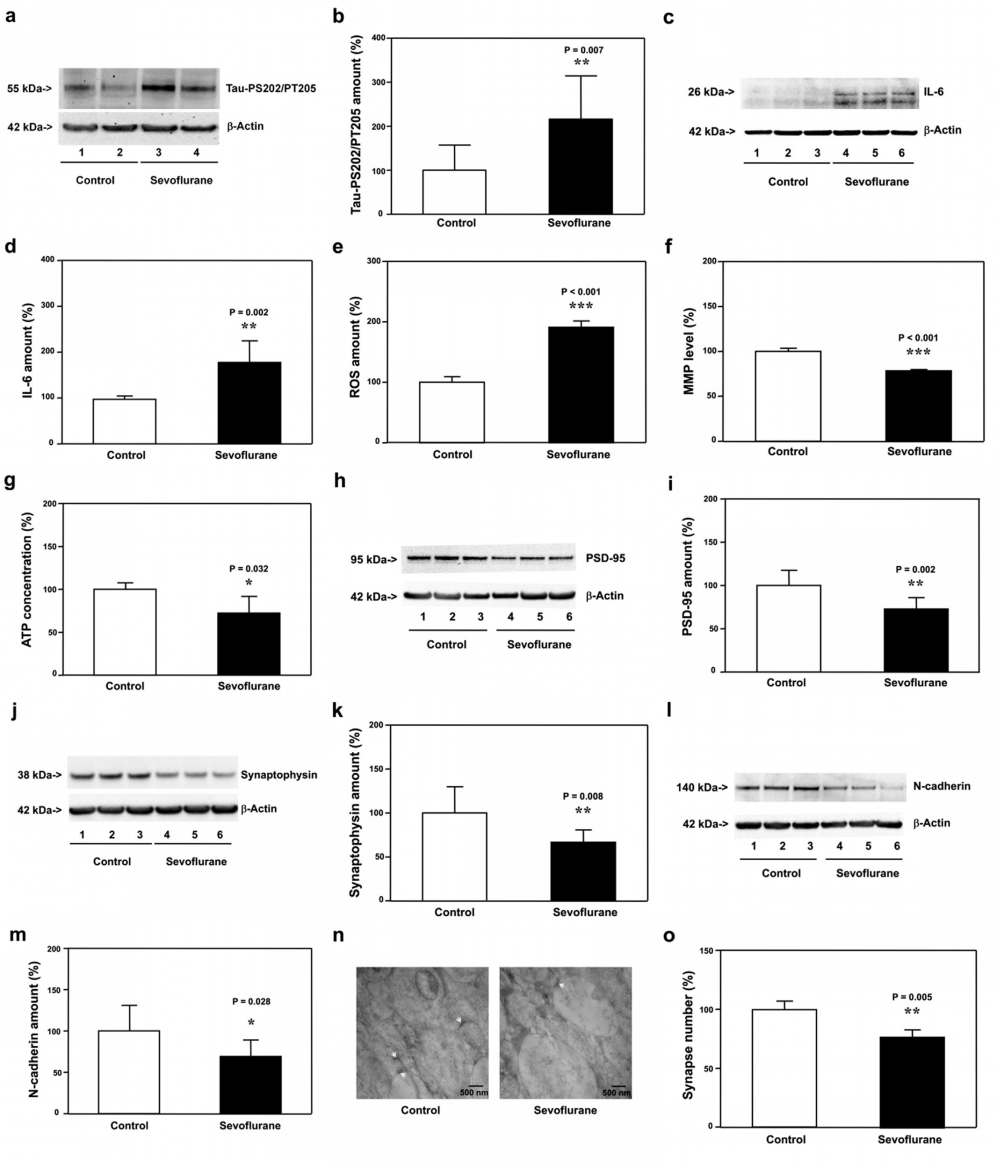
**Effects of sevoflurane on the amounts of pTau, IL-6, ROS, MMP, ATP, PSD-95, synaptophysin, N-cadherin and synapse in the hippocampi of P8 WT mice**. **a.** Sevoflurane (lanes 3 and 4) increased the protein amounts of Tau-PS202/PT205 (detected by antibody AT8) as compared to the control condition (lanes 1 and 2) in the hippocampi of WT mice harvested at P8. **b.** Quantification of the Western blot showed that sevoflurane (black bar) increased the protein amounts of Tau-PS202/PT205 as compared to the control condition (white bar). **c.** Sevoflurane (lanes 4 to 6) increased the protein amounts of IL-6 as compared to the control condition (lanes 1 to 3) in the hippocampi of WT mice harvested at P8. **d.** Quantification of the Western blot showed that sevoflurane (black bar) increased the protein amounts of IL-6 as compared to the control condition (white bar). Sevoflurane (black bar) increased ROS amounts (**e**), decreased MMP amounts (**f**) and reduced ATP amounts (**g**) as compared to the control condition (white bar) in the hippocampi of WT mice harvested at P8. **h**. Sevoflurane (lanes 4 to 6) decreased the protein amounts of PSD-95 as compared to the control condition (lanes 1 to 3) in the hippocampi of WT mice harvested at P8. **i.** Quantification of the Western blot showed that sevoflurane (black bar) decreased the protein amounts of PSD-95 as compared to the control condition (white bar). **j**. Sevoflurane (lanes 4 to 6) reduced the protein amounts of synaptophysin as compared to the control condition (lanes 1 to 3) in the hippocampi of WT mice harvested at P8. **k.** Quantification of the Western blot showed that sevoflurane (black bar) decreased the protein amounts of synaptophysin as compared to the control condition (white bar). **l**. Sevoflurane (lanes 4 to 6) reduced the protein amounts of N-cadherin as compared to the control condition (lanes 1 to 3) in the hippocampi of WT mice harvested at P8. **m.** Quantification of the Western blot showed that sevoflurane (black bar) decreased the protein amounts of N-cadherin as compared to the control condition (white bar). **n**. Sevoflurane reduced the number of the synapse as compared to the control condition in the hippocampi of WT mice harvested at P8. **o.** Quantification of the electron microscope ultrastructure images showed that sevoflurane (black bar) decreased the number of synapses as compared to the control condition (white bar). Tau phosphorylated at serine 202 and threonine 205, Tau-PS202/PT205; phosphorylated Tau, pTau; Interleukin-6, IL-6; Reactive oxygen species, ROS; Mitochondrial membrane potential, MMP; Adenosine triphosphate, ATP; Postsynaptic density 95, PSD-95; Wild-type, WT. N ​= ​9 in each group of the biochemistry studies.

The immunoblotting of Tau-PS202/PT205 showed that sevoflurane increased amounts of Tau-PS202/PT205 in hippocampi of WT mice compared with the control condition at P8, as evidenced by increased visibility of bands representing Tau-PS202/PT205 following sevoflurane than those following the control condition (Fig. 1a). There was no significant difference in amounts of β-Actin in hippocampi of WT P8 mice between the sevoflurane and control condition (Fig. 1a). The quantification of the Western blot, based on the ratio of Tau-PS202/PT205 to β-Actin, indicated that sevoflurane increased amounts of Tau-PS202/PT205 (Fig. 1b, P ​= ​0.007, N ​= ​9). Quantitative Western blot showed that sevoflurane increased amounts of IL-6 in hippocampi of WT mice at P8 (Fig. 1c and d, P ​= ​0.002, N ​= ​9). Sevoflurane increased ROS amounts (Fig. 1e, P ​< ​0.001, N ​= ​9), decreased MMP level (Fig. 1f, P ​< ​0.001, N ​= ​9) and ATP concentrations (Fig. 1g, P ​= ​0.032, N ​= ​9) in hippocampi of WT mice on P8. In addition, sevoflurane decreased amounts of PSD-95 (Fig. 1h and i, P ​= ​0.002, N ​= ​9), synaptophysin (Fig. 1j and k, P ​= ​0.008, N ​= ​9), and N-cadherin (Figure 1l and m, P ​= ​0.028, N ​= ​9) in hippocampi of WT mice on P8. Finally, electron microscope demonstrated that sevoflurane decreased the number of synapses in hippocampi of WT mice on P8 (Fig. 1n and o, P ​= ​0.005, N ​= ​9). These data demonstrated that sevoflurane increased Tau phosphorylation and IL-6 elevation, induced mitochondrial dysfunction and synaptic loss in the hippocampi of WT young mice on P8.

**Fig. 2:**
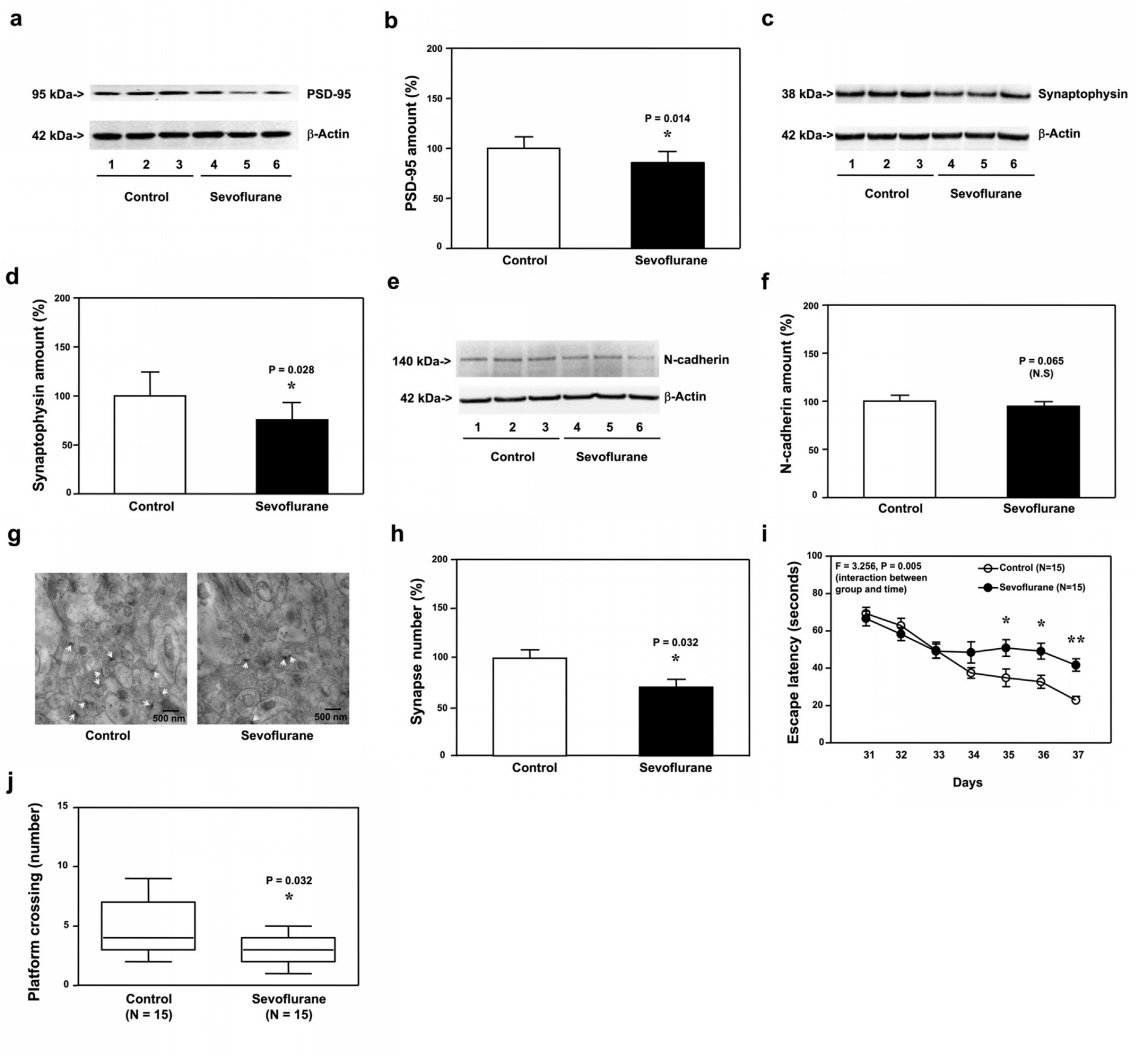
**Effects of sevoflurane on the amounts of PSD-95, synaptophysin, N-cadherin and synapse in the hippocampi of P30 WT mice, and the cognitive function in the mice**. **a.** Sevoflurane (lanes 4 to 6) decreased the protein amounts of PSD-95 as compared to the control condition (lanes 1 to 3) in the hippocampi of WT mice harvested at P30. **b.** Quantification of the Western blot showed that sevoflurane (black bar) decreased the protein amounts of PSD-95 as compared to the control condition (white bar). **c**. Sevoflurane (lanes 4 to 6) reduced the protein amounts of synaptophysin as compared to the control condition (lanes 1 to 3) in the hippocampi of WT mice harvested at P30. **d.** Quantification of the Western blot showed that sevoflurane (black bar) decreased the protein amounts of synaptophysin as compared to the control condition (white bar). **e**. Sevoflurane (lanes 4 to 6) did not apparently change the protein amounts of N-cadherin as compared to the control condition (lanes 1 to 3) in the hippocampi of WT mice harvested at P30. **f.** Quantification of the Western blot showed that sevoflurane (black bar) did not significantly decrease the protein amounts of N-cadherin as compared to the control condition (white bar). **g**. Sevoflurane reduced the number of synapses as compared to the control condition in the hippocampi of WT mice harvested at P30. **h.** Quantification of the electron microscope ultrastructure images showed that sevoflurane (black bar) decreased the number of synapses as compared to the control condition (white bar). **i.** The effects of sevoflurane on the escape latency of the WT mice in MWM from P31 to P37. **j.** The effects of sevoflurane on the number of platform crossing of the WT mice in MWM on P38. Postsynaptic density 95, PSD-95; Morris water maze, MWM; Wild-type, WT. N ​= ​9 in each group of the biochemistry studies and N ​= ​15 in each group of the behavioral studies.

Next, we found that sevoflurane decreased amounts of PSD-95 (Fig. 2a and b, P ​= ​0.014, N ​= ​9) and synaptophysin (Fig. 2c and d, P ​= ​0.028, N ​= ​9), but not N-cadherin (Fig. 2e and f, P ​= ​0.065, N ​= ​9), in hippocampi of WT mice at P30. Moreover, sevoflurane reduced synapse number in hippocampi of WT mice at P30 (Fig. 2g and h, P ​= ​0.032, N ​= ​9). Finally, two-way ANOVA with repeated measurement demonstrated significant interaction of treatment (sevoflurane versus control) and time (P31 to P37) on escape latency in MWM (Fig. 2i, P ​= ​0.005, N ​= ​15). The Student’s t-test with Bonferroni correction showed that sevoflurane specifically increased escape latency as compared to the control condition at P35, P36, and P37. Mann-Whitney-U test showed that the mice had more platform crossing numbers following sevoflurane than those following the control condition at P38 (Fig. 2j, P ​= ​0.032, N ​= ​15). These data indicated that sevoflurane could induce a synaptic loss in hippocampi at P30 and cognitive impairment at P31 to P38 in WT mice. There were not apparent differences in the amounts of pTau and IL-6 between the anesthesia group and control group at P30 (data not shown).

### 3.2 Sevoflurane did not induce IL-6 elevation, mitochondrial dysfunction, synaptic loss and cognitive impairment in Tau KO young mice

**Fig. 3:**
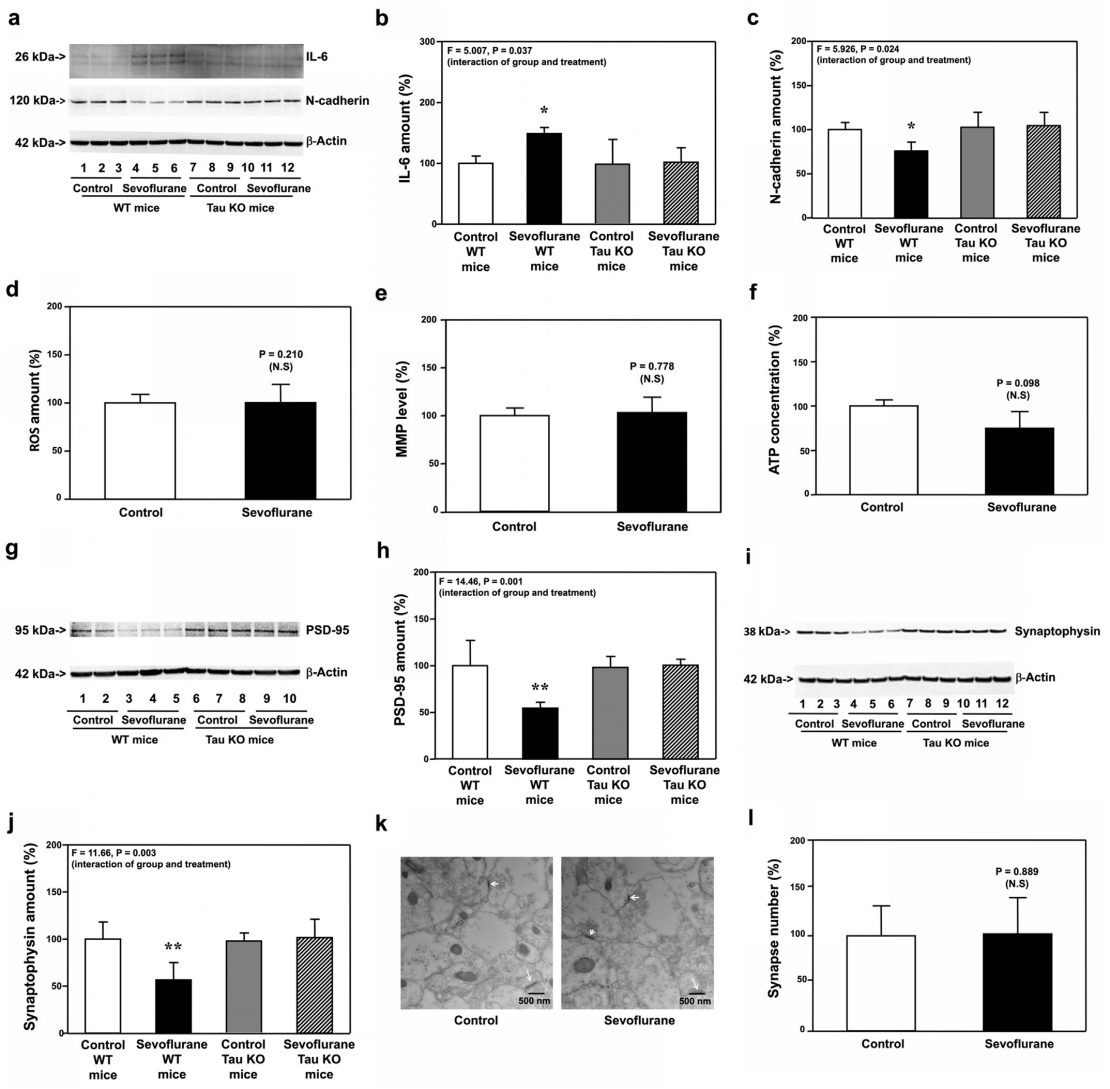
**Effects of sevoflurane on the amounts of IL-6, ROS, MMP, ATP, PSD-95, synaptophysin, N-cadherin and synapse in the hippocampi of P8 Tau KO mice**. **a.** Sevoflurane (lanes 4 to 6) increased the protein amounts of IL-6 and decreased the protein amounts of N-cadherin as compared to the control condition (lanes 1 to 3) in the hippocampi of WT mice harvested at P8. In the Tau KO mice, however, sevoflurane (lanes 10 to 12) did not apparently change the amounts of IL-6 and N-cadherin as compared to the control condition (lanes 7 to 9) in the hippocampi of the Tau KO mice harvested at P8. **b.** Quantification of the Western blot showed that sevoflurane (black bar) increased the protein amounts of IL-6 as compared to the control condition (white bar) in the WT mice. However, sevoflurane (stripe bar) did not significantly increase the protein amounts of IL-6 as compared to the control condition (gray bar) in the Tau KO mice. **c**. Quantification of the Western blot also showed that sevoflurane (black bar) decreased the protein amounts of N-cadherin as compared to the control condition (white bar) in the WT mice. However, sevoflurane (stripe bar) did not decrease the protein amounts of N-cadherin as compared to the control condition (gray bar) in the hippocampi of Tau KO mice harvested at P8. Sevoflurane (black bar) did not significantly change the amounts of ROS (**d**), MMP (**e**) and ATP (**f**) as compared to the control condition (white bar) in the hippocampi of Tau KO mice harvested at P8. **g.** Sevoflurane (lanes 3 to 5) decreased the protein amounts of PSD-95 as compared to the control condition (lanes 1 to 2) in the hippocampi of WT mice harvested at P8. In the Tau KO mice, however, sevoflurane (lanes 9 to 10) did not significantly change the protein amounts of PSD-95 as compared to the control condition (lanes 6 to 8) in the hippocampi of the Tau KO mice harvested at P8. **h**. Quantification of the Western blot showed that sevoflurane (black bar) decreased the protein amounts of PSD-95 as compared to the control condition (white bar) in the WT mice. However, sevoflurane (stripe bar) did not decrease the protein amounts of PSD-95 as compared to the control condition (gray bar) in the Tau KO mice. **i**. Sevoflurane (lanes 4 to 6) decreased the protein amounts of synaptophysin as compared to the control condition (lanes 1 to 3) in the hippocampi of WT mice harvested at P8. In the Tau KO mice, however, sevoflurane (lanes 10 to 12) did not apparently change the protein amounts of synaptophysin as compared to the control condition (lanes 7 to 9) in the hippocampi of the Tau KO mice harvested at P8. **j**. Quantification of the Western blot showed that sevoflurane (black bar) decreased the protein amounts of synaptophysin as compared to the control condition (white bar) in the WT mice. But sevoflurane (stripe bar) did not significantly decrease the protein amounts of synaptophysin as compared to the control condition (gray bar) in the hippocampi of Tau KO mice harvested at P8. **k**. Sevoflurane did not significantly change the number of synapses as compared to the control condition in the hippocampi of Tau KO mice harvested at P8. **l.** Quantification of the electron microscope ultrastructure images showed that sevoflurane (black bar) did not significantly decrease the number of synapses as compared to the control condition (white bar) in the hippocampi of Tau KO mice. Interleukin-6, IL-6; Reactive oxygen species, ROS; Mitochondrial membrane potential, MMP; Adenosine triphosphate, ATP; Postsynaptic density 95, PSD-95; Wild type, WT; Knockout, KO. N ​= ​9 in each group of the biochemistry studies.

**Fig. 4:**
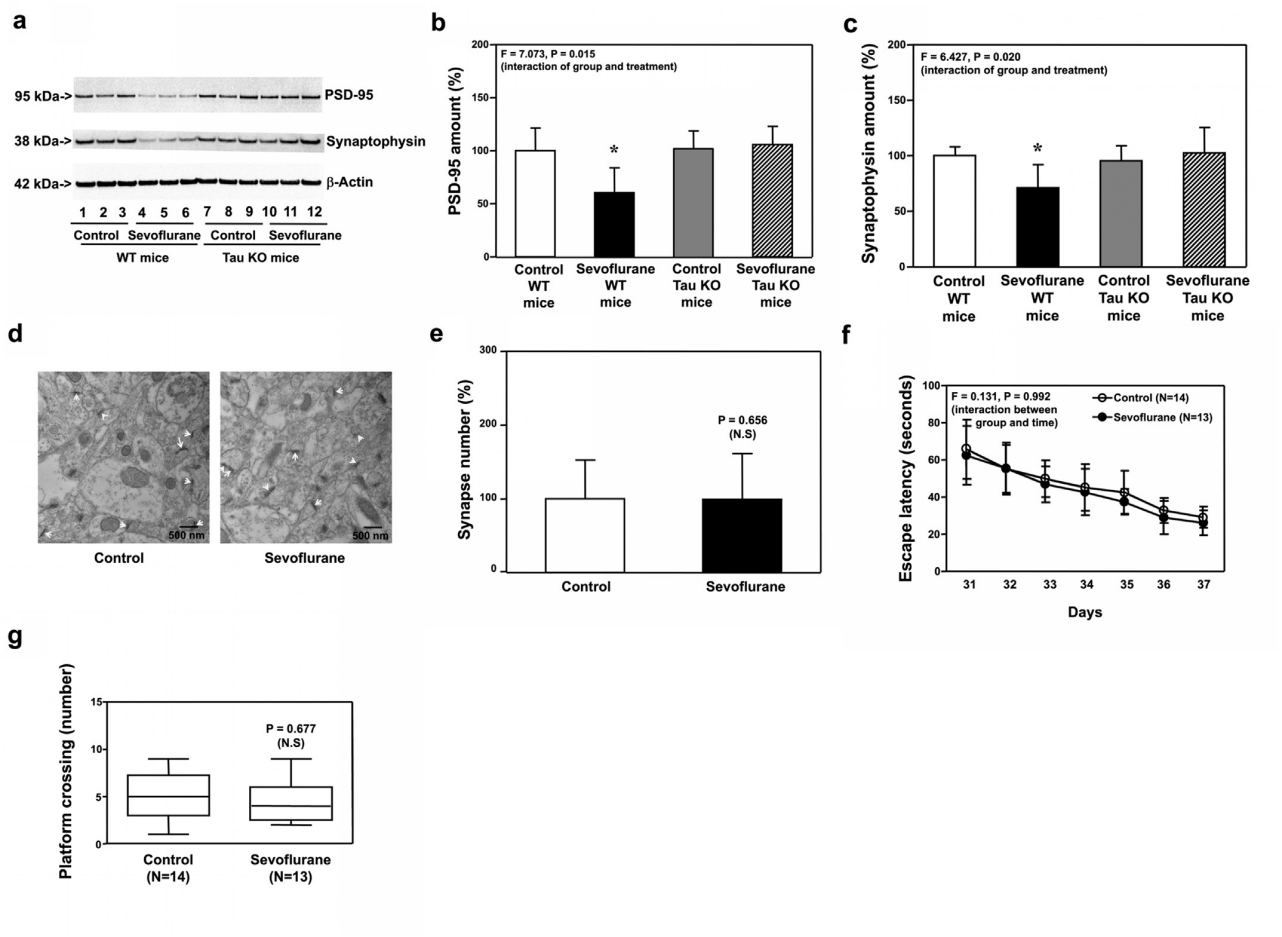
**Effects of sevoflurane on the amounts of PSD-95, synaptophysin, and synapse in the hippocampi of P30 Tau KO mice, and the cognitive function in the mice**. **a.** Sevoflurane (lanes 4 to 6) decreased the protein amounts of PSD-95 and synaptophysin as compared to the control condition (lanes 1 to 3) in the hippocampi of WT mice harvested at P30. In the Tau KO mice, however, sevoflurane (lanes 10–12) did not significantly change the protein amounts of PSD-95 and synaptophysin as compared to the control condition (lanes 7 to 9) in the hippocampi of Tau KO mice harvested at P30. **b.** Quantification of the Western blot showed that sevoflurane (black bar) decreased the protein amounts of PSD-95 as compared to the control condition (white bar) in the hippocampi of WT mice harvested at P30. In the Tau KO mice, however, sevoflurane (stripe bar) did not apparently change the amounts of PSD-95 as compared to the control condition (gray bar) in the hippocampi of Tau KO mice harvested at P30. **c.** Quantification of the Western blot showed that sevoflurane (black bar) decreased the protein amounts of synaptophysin as compared to the control condition (white bar) in the hippocampi of WT mice harvested at P30. In the Tau KO mice, however, sevoflurane (stripe bar) did not significantly change the amounts of synaptophysin as compared to the control condition (gray bar) in the hippocampi of Tau KO mice harvested at P30. **d**. Sevoflurane did not decrease the number of synapses as compared to the control condition in the hippocampi of Tau KO mice harvested at P30. **e.** Quantification of the electron microscope ultrastructure images showed that sevoflurane (black bar) did not decrease the number of the synapse as compared to the control condition (white bar) in Tau KO mice. **f.** The effects of sevoflurane on the escape latency of the Tau KO mice in MWM from P31 to P37. **g.** The effects of sevoflurane on the platform crossing number of the Tau KO mice in MWM on P38. Postsynaptic density 95, PSD-95; Morris Water Maze, MWM; Wild type, WT; Knock out, KO. N ​= ​9 in each group of the biochemistry studies and N ​= ​13–14 in each group of the behavioral studies.

We found that sevoflurane induced neither IL-6 elevation, mitochondrial dysfunction nor synaptic loss in hippocampi of Tau KO mice harvested on P8 (Fig. 3). Moreover, sevoflurane did not cause synaptic loss and cognitive impairment in Tau KO mice on P31 to P38 (Fig. 4). These data indicated that IL-6 elevation, mitochondrial dysfunction, synaptic loss and cognitive impairment in the young mice were dependent on Tau protein.

### 3.3 Sevoflurane increased Tau phosphorylation, but did not induce mitochondrial dysfunction, synaptic loss and cognitive impairment, in IL-6 KO young mice

**Fig. 5:**
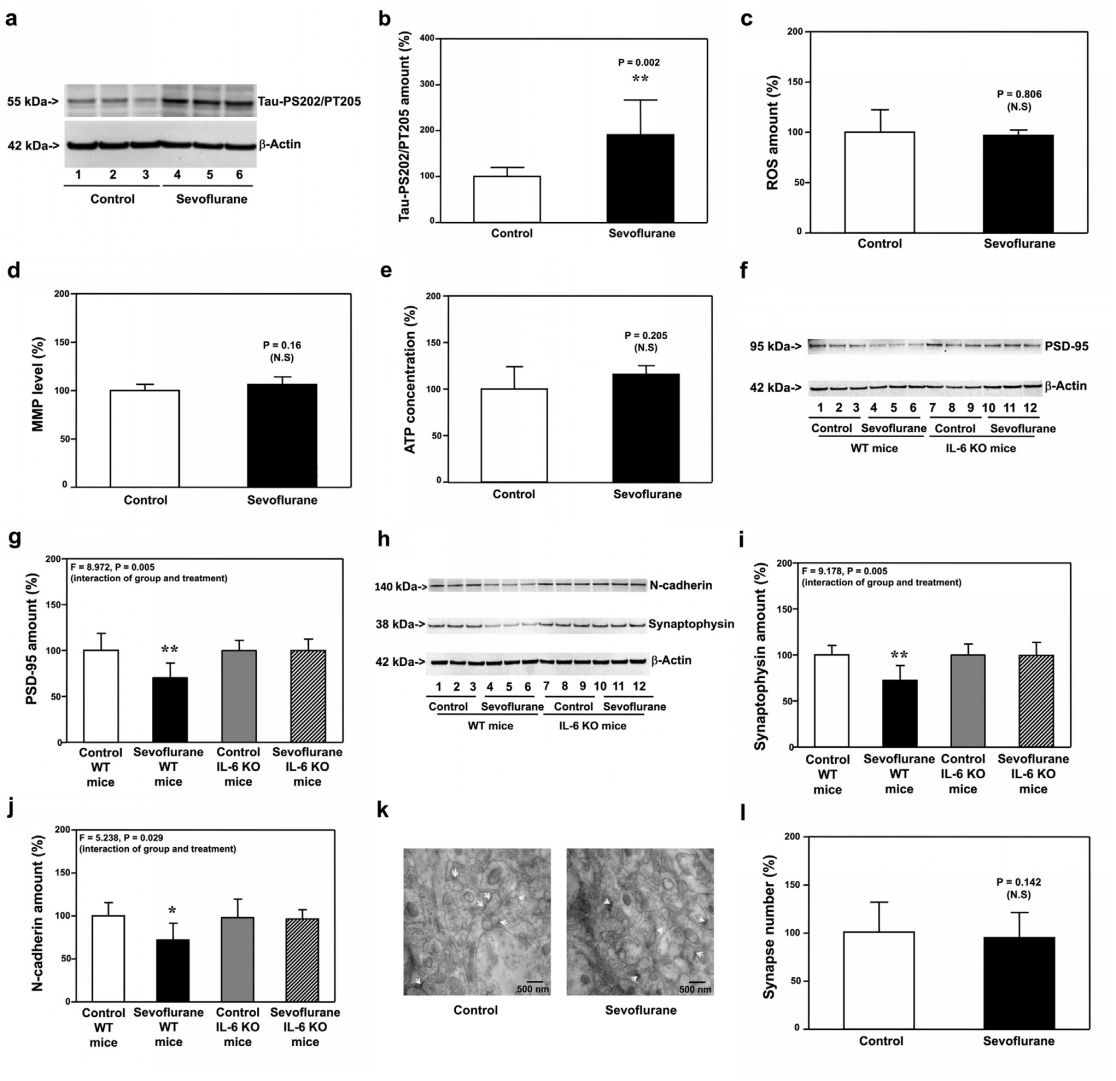
**Effects of sevoflurane on the amounts of pTau, ROS, MMP, ATP, PSD-95, synaptophysin, N-cadherin and synapse in the hippocampi of P8 IL-6 KO mice**. **a.** Sevoflurane (lanes 4 to 6) increased the protein amounts of Tau-PS202/PT205 as compared to the control condition (lanes 1 to 3) in the hippocampi of IL-6 KO mice harvested at P8. **b.** Quantification of the Western blot showed that sevoflurane (black bar) increased the protein amounts of Tau-PS202/PT205 as compared to the control condition (white bar) in the IL-6 KO mice harvested at P8. Sevoflurane (black bar) did not significantly change the amounts of ROS (**c**), MMP (**d**) and ATP (**e**) as compared to the control condition (white bar) in the hippocampi of IL-6 KO mice harvested at P8. **f.** Sevoflurane (lanes 4 to 6) decreased the protein amounts of PSD-95 as compared to the control condition (lanes 1 to 3) in the hippocampi of WT mice harvested at P8. In the IL-6 KO mice, however, sevoflurane (lanes 10 to 12) did not significantly change the protein amounts of PSD-95 as compared to the control condition (lanes 7 to 9) in the hippocampi of the IL-6 KO mice harvested at P8. **g**. Quantification of the Western blot showed that sevoflurane (black bar) decreased the protein amounts of PSD-95 as compared to the control condition (white bar) in the WT mice. However, sevoflurane (stripe bar) did not decrease the protein amounts of PSD-95 as compared to the control condition (gray bar) in the IL-6 KO mice. **h**. Sevoflurane (lanes 4 to 6) decreased the protein amounts of synaptophysin and N-cadherin as compared to the control condition (lanes 1 to 3) in the hippocampi of WT mice harvested at P8. In the IL-6 KO mice, however, sevoflurane (lanes 10 to 12) did not significantly change the protein amounts of synaptophysin and N-cadherin as compared to the control condition (lanes 7 to 9) in the hippocampi of the IL-6 KO mice harvested at P8. **i**. Quantification of the Western blot showed that sevoflurane (black bar) decreased the protein amounts of synaptophysin as compared to the control condition (white bar) in the WT mice. However, sevoflurane (stripe bar) did not significantly decrease the protein amounts of synaptophysin as compared to the control condition (gray bar) in the IL-6 KO mice. **j**. Quantification of the Western blot showed that sevoflurane (black bar) decreased the protein amounts of N-cadherin as compared to the control condition (white bar) in the WT mice. However, sevoflurane (stripe bar) did not significantly decrease the protein amounts of N-cadherin as compared to the control condition (gray bar) in the IL-6 KO mice. **k**. Sevoflurane did not significantly change the number of synapses as compared to the control condition in the hippocampi of IL-6 KO mice harvested at P8. **l**. Quantification of the electron microscope ultrastructure images showed that sevoflurane (black bar) did not significantly change the number of synapses as compared to the control condition (white bar) in IL-6 KO mice. Tau phosphorylated at serine 202 and threonine 205, Tau-PS202/PT205; phosphorylated Tau, pTau; Interleukin-6, IL-6; Reactive oxygen species, ROS; Mitochondrial membrane potential, MMP; Adenosine triphosphate, ATP; Postsynaptic density 95, PSD-95; Wild type, WT; Knock out, KO. N ​= ​9 in each group of the biochemistry studies.

Next, we found that sevoflurane still increased Tau phosphorylation in hippocampi of IL-6 KO mice at P8 (Fig. 5a and b, P ​= ​0.002, N ​= ​9). However, sevoflurane did not induce mitochondrial dysfunction (Fig. 5c, d, and e) in hippocampi of P8 IL-6 KO mice. In addition, sevoflurane still decreased amounts of PSD-95 (Fig. 5f and g, P ​= ​0.005, N ​= ​9), synaptophysin (Fig. 5h and i, P ​= ​0.005, N ​= ​9), and N-cadherin (Fig. 5h and j, P ​= ​0.029, N ​= ​9) in hippocampi of P8 WT mice, but sevoflurane did not reduce amounts of these proteins (Fig. 5f, g, h, i, and j) in hippocampi of P8 IL-6 KO mice. Finally, sevoflurane did not reduce synapse number in hippocampi of P8 IL-6 KO mice (Fig. 5k and l).

**Fig. 6:**
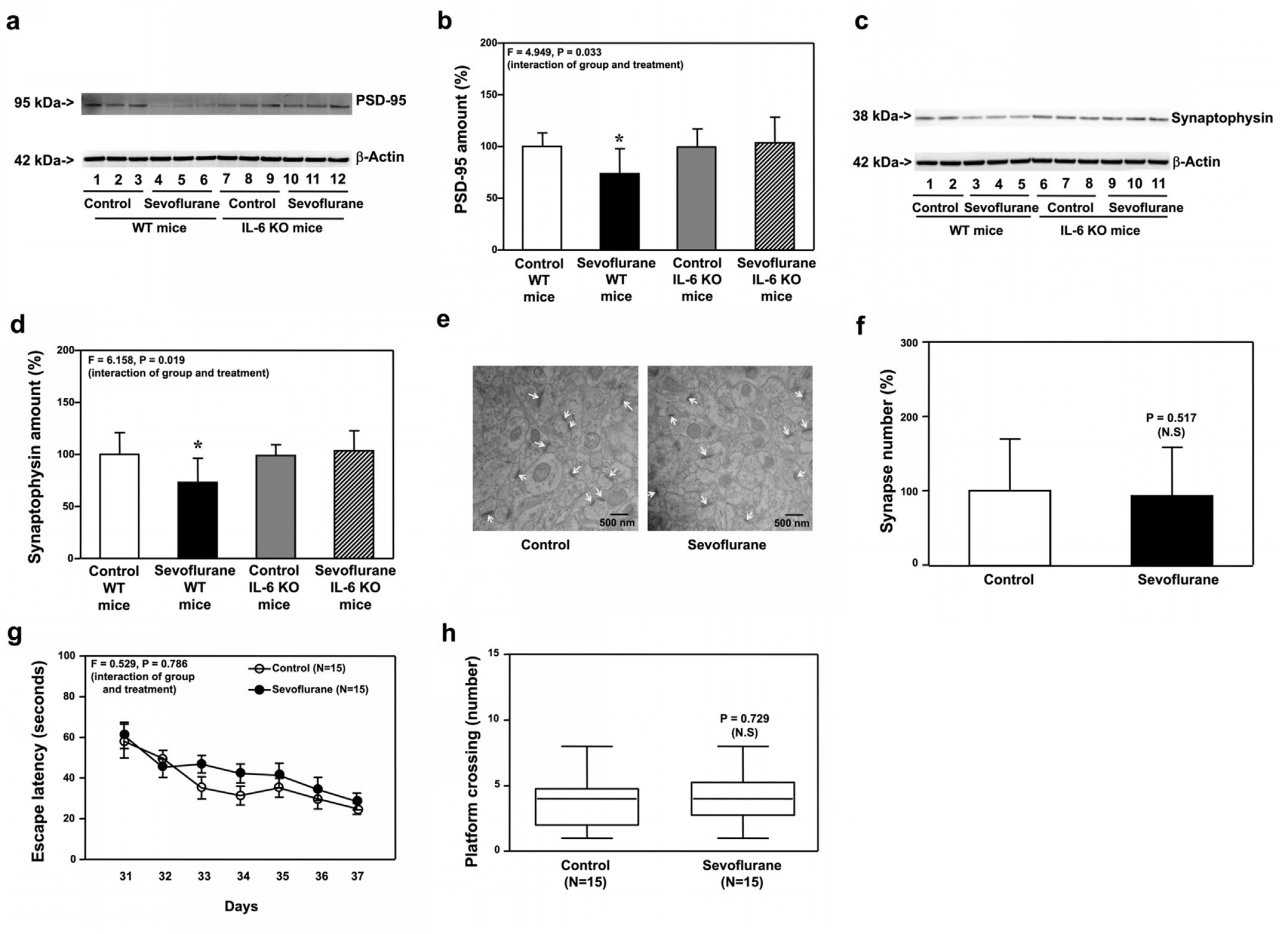
**Effects of sevoflurane on the amounts of PSD-95, synaptophysin, N-cadherin and synapse in the hippocampi of P30 IL-6 KO mice, and the cognitive function in the mice**. **a.** Sevoflurane (lanes 4 to 6) decreased the protein amounts of PSD-95 as compared to the control condition (lanes 1 to 3) in the hippocampi of WT mice harvested at P30. In the IL-6 KO mice, however, sevoflurane (lanes 10 to 12) did not apparently change the protein amounts of PSD-95 as compared to the control condition (lanes 7 to 9) in the hippocampi of the IL-6 KO mice harvested at P30. **b.** Quantification of the Western blot showed that sevoflurane (black bar) decreased the protein amounts of PSD-95 as compared to the control condition (white bar) in the hippocampi of WT mice harvested at P30. In the IL-6 KO mice, however, sevoflurane (stripe bar) did not significantly change the protein amounts of PSD-95 as compared to the control condition (gray bar) in the hippocampi of the IL-6 KO mice harvested at P30. **c**. Sevoflurane (lanes 3 to 5) decreased the protein amounts of synaptophysin as compared to the control condition (lanes 1 to 2) in the hippocampi of WT mice harvested at P30. In the IL-6 KO mice, however, sevoflurane (lanes 9 to 11) did not apparently change the protein amounts of synaptophysin as compared to the control condition (lanes 6 to 8) in the hippocampi of the IL-6 KO mice harvested at P30. **d**. Quantification of the Western blot showed that sevoflurane (black bar) decreased the protein amounts of synaptophysin as compared to the control condition (white bar) in the hippocampi of WT mice harvested at P30. In the IL-6 KO mice, however, sevoflurane (stripe bar) did not significantly change the amounts of synaptophysin as compared to the control condition (gray bar) in the hippocampi of the IL-6 KO mice harvested at P30. **e**. Sevoflurane did not decrease the number of synapses as compared to the control condition in the hippocampi of the IL-6 KO mice harvested at P30. **f.** Quantification of the electron microscope ultrastructure images showed that sevoflurane (black bar) did not significantly change the number of synapses as compared to the control condition (white bar) in the IL-6 KO mice. **g.** The effects of sevoflurane on the escape latency of the IL-6 KO mice in MWM from P31 to P37. **h.** The effects of sevoflurane on the platform crossing number of the IL-6 KO mice in MWM on P38. Postsynaptic density 95, PSD-95. Morris Water Maze, MWM; Wild type, WT; Knockout, KO. N ​= ​9 in each group of the biochemistry studies and N ​= ​15 in each group of the behavioral studies.

Moreover, sevoflurane did not reduce amounts of PSD-95 (Fig. 6a and b) and synaptophysin (Fig. 6c and d) in hippocampi of IL-6 KO mice at P30, while sevoflurane still decreased amounts of PSD-95 (Fig. 6a and b, P ​= ​0.033, N ​= ​9) and synaptophysin (Fig. 6c and d, P ​= ​0.019, N ​= ​9) in hippocampi of WT mice at P30. Sevoflurane did not reduce synapse number in hippocampi of IL-6 KO mice at P30 (Fig. 6e and f). Finally, sevoflurane did not cause cognitive impairment in IL-6 KO mice from P31 to P38 (Fig. 6g and h).

Taken together, these data, obtained from both Tau KO (Fig. 3, Fig. 4) and IL-6 KO (Fig. 5, Fig. 6) mice, indicated that IL-6 elevation in hippocampi of young mice was dependent on Tau, but independent of mitochondrial dysfunction, synaptic loss and cognitive impairment in young mice.

### 3.4 Sevoflurane induced increase in Tau phosphorylation, IL-6 elevation, but not mitochondrial dysfunction, synaptic loss and cognitive impairment, in CypD KO young mice

**Fig. 7:**
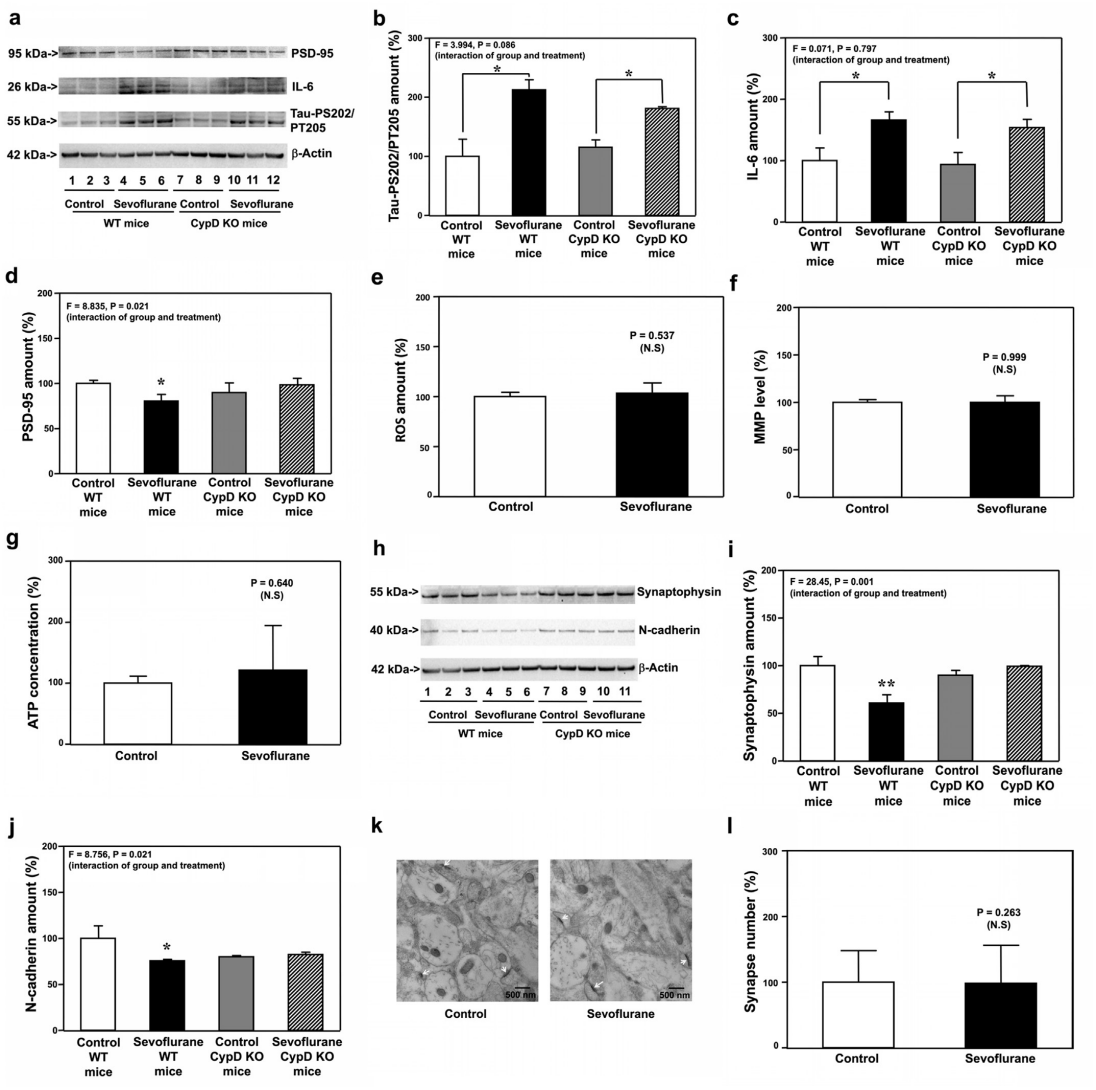
**Effects of sevoflurane on the amounts of pTau, IL-6, ROS, MMP, ATP, PSD-95, synaptophysin, N-cadherin and synapse in the hippocampi of P8 CypD KO mice**. **a.** Sevoflurane (lanes 4 to 6) increased the protein amounts of Tau-PS202/PT205 and IL-6 but decreased the protein amounts of PSD-95 as compared to the control condition (lanes 1 to 3) in the hippocampi of WT mice harvested at P8. In the CypD KO mice, however, sevoflurane (lanes 10 to 12) still increased the protein amounts of Tau-PS202/PT205 and IL-6 but did not significantly change the protein amounts of PSD-95 as compared to the control condition (lanes 7 to 9) in the hippocampi of the CypD KO mice harvested at P8. Quantification of the Western blot showed that sevoflurane (black bar) increased the protein amounts of Tau-PS202/PT205 (**b**) and IL-6 (**c**) as compared to the control condition (white bar) in the hippocampi of the WT mice harvested at P8. In the CypD KO mice, however, sevoflurane (stripe bar) still increased the protein amounts of Tau-PS202/PT205 (**b**) and IL-6 (**c**) as compared to the control condition (gray bar) in the hippocampi of the CypD KO mice harvested at P8. **d**. Quantification of the Western blot showed that sevoflurane (black bar) decreased the protein amounts of PSD-95 as compared to the control condition (white bar) in the hippocampi of the WT mice harvested at P8. However, sevoflurane (stripe bar) did not significantly change the protein amounts of PSD-95 as compared to the control condition (gray bar) in the hippocampi of the CypD KO mice harvested at P8. Sevoflurane (black bar) did not significantly change the amounts of ROS (**e**), MMP (**f**) and ATP (**g**) as compared to the control condition (white bar) in the hippocampi of CypD KO mice harvested at P8. **h.** Sevoflurane (lanes 4 to 6) decreased the protein amounts of synaptophysin and N-cadherin as compared to the control condition (lanes 1 to 3) in the hippocampi of WT mice harvested at P8. In the CypD KO mice, however, sevoflurane (lanes 10 to 11) did not significantly change the protein amounts of synaptophysin and N-cadherin as compared to the control condition (lanes 7 to 9) in the hippocampi of the CypD KO mice harvested at P8. Quantification of the Western blot showed that sevoflurane (black bar) decreased the protein amounts of synaptophysin (**i**) and N-cadherin (**j**) as compared to the control condition (white bar) in the hippocampi of the WT mice harvested at P8. In the CypD KO mice, however, sevoflurane (stripe bar) did not significantly change the protein amounts of synaptophysin (**i**) and N-cadherin (**j**) as compared to the control condition (gray bar) in the hippocampi of the CypD KO mice harvested at P8. **k**. Sevoflurane did not significantly change the number of synapses as compared to the control condition in the hippocampi of CypD KO mice harvested at P8. **l**. Quantification of the electron microscope ultrastructure images showed that sevoflurane (black bar) did not significantly change the number of synapses as compared to the control condition (white bar) in the CypD KO mice. Tau phosphorylated at serine 202 and threonine 205, Tau-PS202/PT205; phosphorylated Tau, pTau; Interleukin-6, IL-6; Postsynaptic density 95, PSD-95; Cyclophilin-D, CypD; Reactive oxygen species, ROS; Mitochondrial membrane potential, MMP; Adenosine triphosphate, ATP; Wild-type, WT; Knockout, KO. N ​= ​9 in each group of the biochemistry studies.

We found that sevoflurane increased Tau phosphorylation (Fig. 7a and b) and IL-6 elevation (Fig. 7a and c) in hippocampi of both WT mice and CypD KO mice at P8. However, sevoflurane reduced protein amounts of PSD-95 in hippocampi of P8 WT, but not P8 CypD KO, mice (Fig. 7a and d, P ​= ​0.021, N ​= ​9). Similarly, sevoflurane reduced the amounts of synaptophysin (Fig. 7h and i, P ​= ​0.001, N ​= ​9) and N-cadherin (Fig. 7h and j, P ​= ​0.021, N ​= ​9) in hippocampi of P8 WT, but not P8 CypD KO, mice. Sevoflurane induced neither mitochondrial dysfunction (Fig. 7e, f, and g) nor synaptic loss (Fig. 7k and l) in hippocampi of CypD KO mice on P8.

**Fig. 8:**
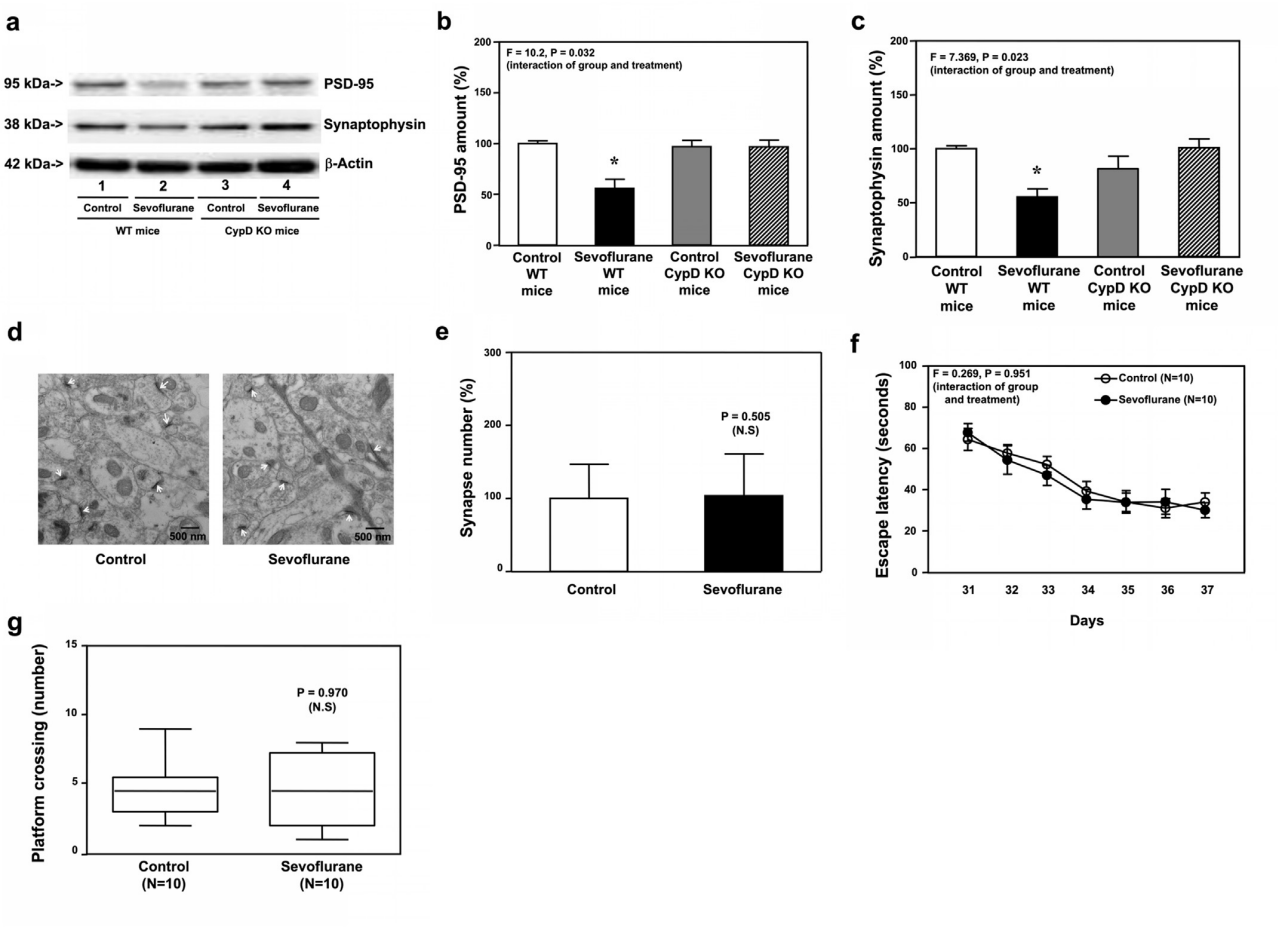
**Effects of sevoflurane on the amounts of PSD-95, synaptophysin, and synapse in the hippocampi of P30 CypD KO mice, and the cognitive function in the mice**. **a.** Sevoflurane (lane 2) decreased the protein amounts of PSD-95 as compared to the control condition (lane 1) in the hippocampi of WT mice harvested at P30. In the CypD KO mice, however, sevoflurane (lane 4) did not apparently change the protein amounts of PSD-95 as compared to the control condition (lane 3) in the hippocampi of the CypD KO mice harvested at P30. Quantification of the Western blot showed that sevoflurane (black bar) decreased the protein amounts of PSD-95 (**b**) and synaptophysin (**c**) as compared to the control condition (white bar) in the hippocampi of WT mice harvested at P30. In the CypD KO mice, however, sevoflurane (stripe bar) did not significantly change the amounts of PSD-95 (**b**) and synaptophysin (**c**) as compared to the control condition (gray bar) in the hippocampi of the CypD KO mice harvested at P30. **d**. Sevoflurane did not decrease the number of synapses as compared to the control condition in the hippocampi of the CypD KO mice harvested at P30. **e**. Quantification of the electron microscope ultrastructure images showed that sevoflurane (black bar) did not significantly change the number of synapses as compared to the control condition (white bar) in the CypD KO mice. **f**. The effects of sevoflurane on the escape latency of the CypD KO mice in MWM from P31 to P37. **g**. The effects of sevoflurane on the platform crossing number of the CypD KO mice in MWM on P38. Postsynaptic density 95, PSD-95; Morris Water Maze, MWM; Wild type, WT; Knock out, KO. N ​= ​9 in each group of the biochemistry studies and N ​= ​15 in each group of the behavioral studies.

Sevoflurane reduced amounts of PSD-95 (Fig. 8a and b, P ​= ​0.032, N ​= ​9) and synaptophysin (Fig. 8a and c, P ​= ​0.023, N ​= ​9) in hippocampi of P30 WT, but not P30 CypD KO, mice. Sevoflurane did not reduce synapse number in hippocampi of CypD KO mice at P30 (Fig. 8d and e). Finally, sevoflurane did not cause cognitive impairment in CypD KO mice tested from P31 to P37 (Fig. 8f and g).

These data, obtained from Tau KO mice (Fig. 3, Fig. 4), IL-6 KO (Fig. 5, Fig. 6) and CypD KO (Fig. 7, Fig. 8) mice, indicated the mitochondrial dysfunction in young mice was dependent on Tau and IL-6, but independent of synaptic loss and cognitive impairment, in young mice.

### *3.5* Idebenone attenuated sevoflurane-induced cognitive impairment in young mice

**Fig. 9:**
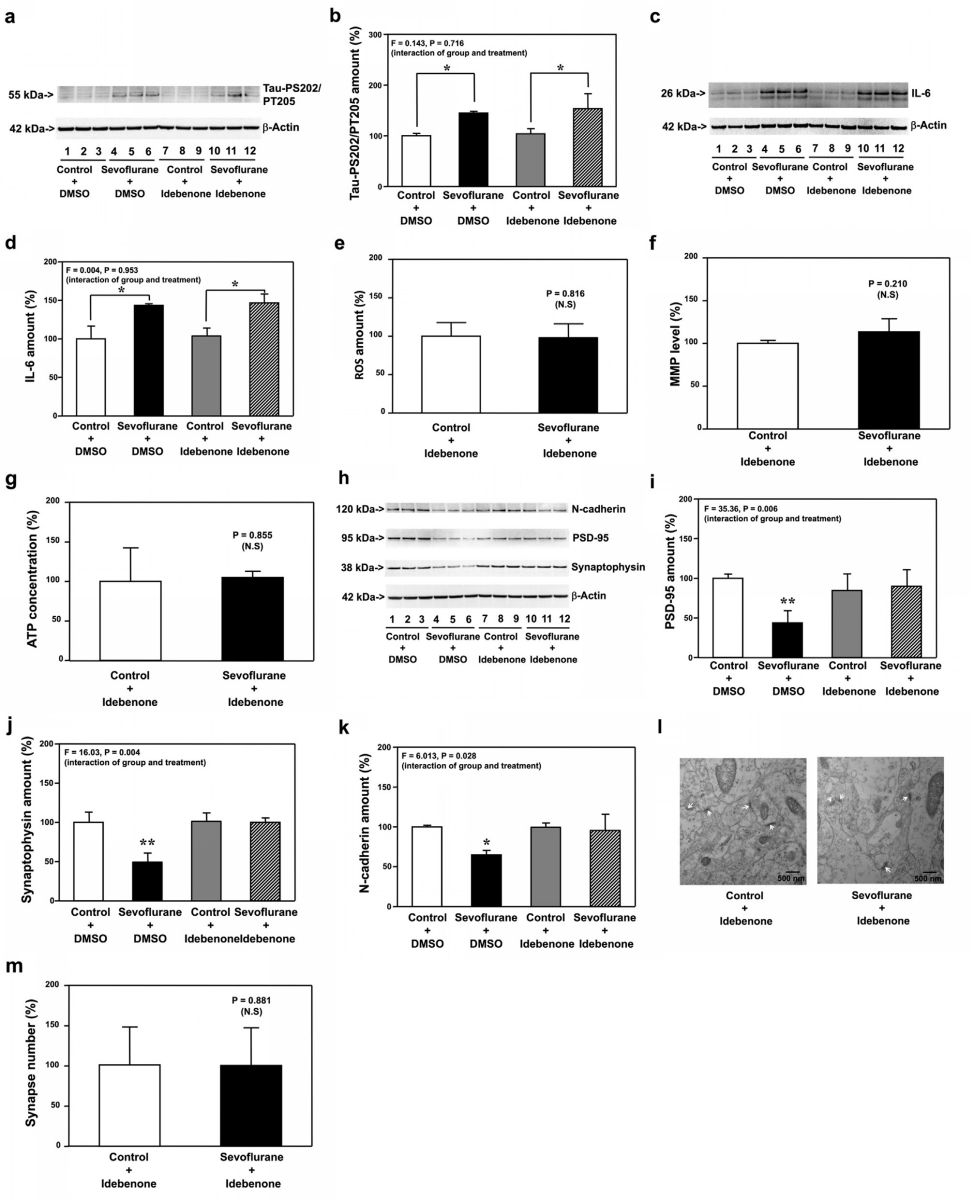
**Effects of idebenone on sevoflurane-induced changes in the P8 WT mice**. Sevoflurane (lanes 4 to 6) increased the protein amounts of Tau-PS202/PT205 (**a**) and IL-6 (**c**) as compared to the control condition (lanes 1 to 3) in the hippocampi of WT mice harvested at P8. In the WT P8 mice pretreated with idebenone, however, sevoflurane (lanes 10 to 12) still increased the protein amounts of Tau-PS202/PT205 (**a**) and IL-6 (**c**) as compared to the control condition (lanes 7 to 9) in the hippocampi of the WT mice pretreated with idebenone harvested at P8. Quantification of the Western blot showed that sevoflurane (black bar) increased the protein amounts of Tau-PS202/PT205 (**b**) and IL-6 (**d**) as compared to the control condition (white bar) in the hippocampi of the WT mice harvested at P8. In the WT mice pretreated with idebenone, sevoflurane (stripe bar) still increased the protein amounts of Tau-PS202/PT205 (**b**) and IL-6 (**d**) as compared to the control condition (gray bar) in the hippocampi of the WT mice harvested at P8. Sevoflurane (black bar) did not significantly change the amounts of ROS (**e**), MMP (**f**) and ATP (**g**) as compared to the control condition (white bar) in the hippocampi of the WT mice pretreated with idebenone harvested at P8. **h.** Sevoflurane (lanes 4 to 6) decreased the protein amounts of PSD-95, synaptophysin, and N-cadherin as compared to the control condition (lanes 1 to 3) in the hippocampi of WT mice harvested at P8. In the WT mice pretreated with idebenone, however, sevoflurane (lanes 10 to 12) did not apparently change the protein amounts of PSD-95, synaptophysin, and N-cadherin as compared to the control condition (lanes 7 to 9) in the hippocampi of the WT mice pretreated with idebenone harvested at P8. Quantification of the Western blot showed that sevoflurane (black bar) decreased the protein amounts of PSD-95 (**i**), synaptophysin (**j**) and N-cadherin (**k**) as compared to the control condition (white bar) in the hippocampi of the WT mice harvested at P8. In the WT mice pretreated with idebenone, however, sevoflurane (stripe bar) did not significantly change the protein amounts of PSD-95 (**i**), synaptophysin (**j**) and N-cadherin (**k**) as compared to the control condition (gray bar) in the hippocampi of the WT mice pretreated with idebenone harvested at P8. **l**. Sevoflurane did not significantly change the number of synapses as compared to the control condition in the hippocampi of WT mice pretreated with idebenone harvested at P8. **m.** Quantification of the electron microscope ultrastructure images showed that sevoflurane (black bar) did not significantly change the number of synapses as compared to the control condition (white bar) in WT mice pretreated with idebenone. Tau phosphorylated at serine 202 and threonine 205, Tau-PS202/PT205; Interleukin-6, IL-6; Reactive oxygen species, ROS; Mitochondrial membrane potential, MMP; Adenosine triphosphate, ATP; Postsynaptic density 95, PSD-95; Wild-type, WT. N ​= ​9 in each group of the biochemistry studies.

We found that idebenone mitigated neither sevoflurane-increased Tau phosphorylation (Fig. 9a and b) nor sevoflurane-induced IL-6 elevation (Fig. 9c and d). But, sevoflurane did not induce mitochondrial dysfunction in the WT mice pretreated with idebenone (Fig. 9e, f, and g). Moreover, idebenone mitigated the sevoflurane-induced reduction in PSD-95 (Fig. 9h and i, P ​= ​0.006, N ​= ​9), synaptophysin (Fig. 9h and j, P ​= ​0.004, N ​= ​9) and N-cadherin (Fig. 9h and k, P ​= ​0.028, N ​= ​9) in hippocampi of WT mice at P8. Finally, sevoflurane did not reduce synapse number in P8 WT mice pretreated with idebenone (Figure 9l and m).

**Fig. 10:**
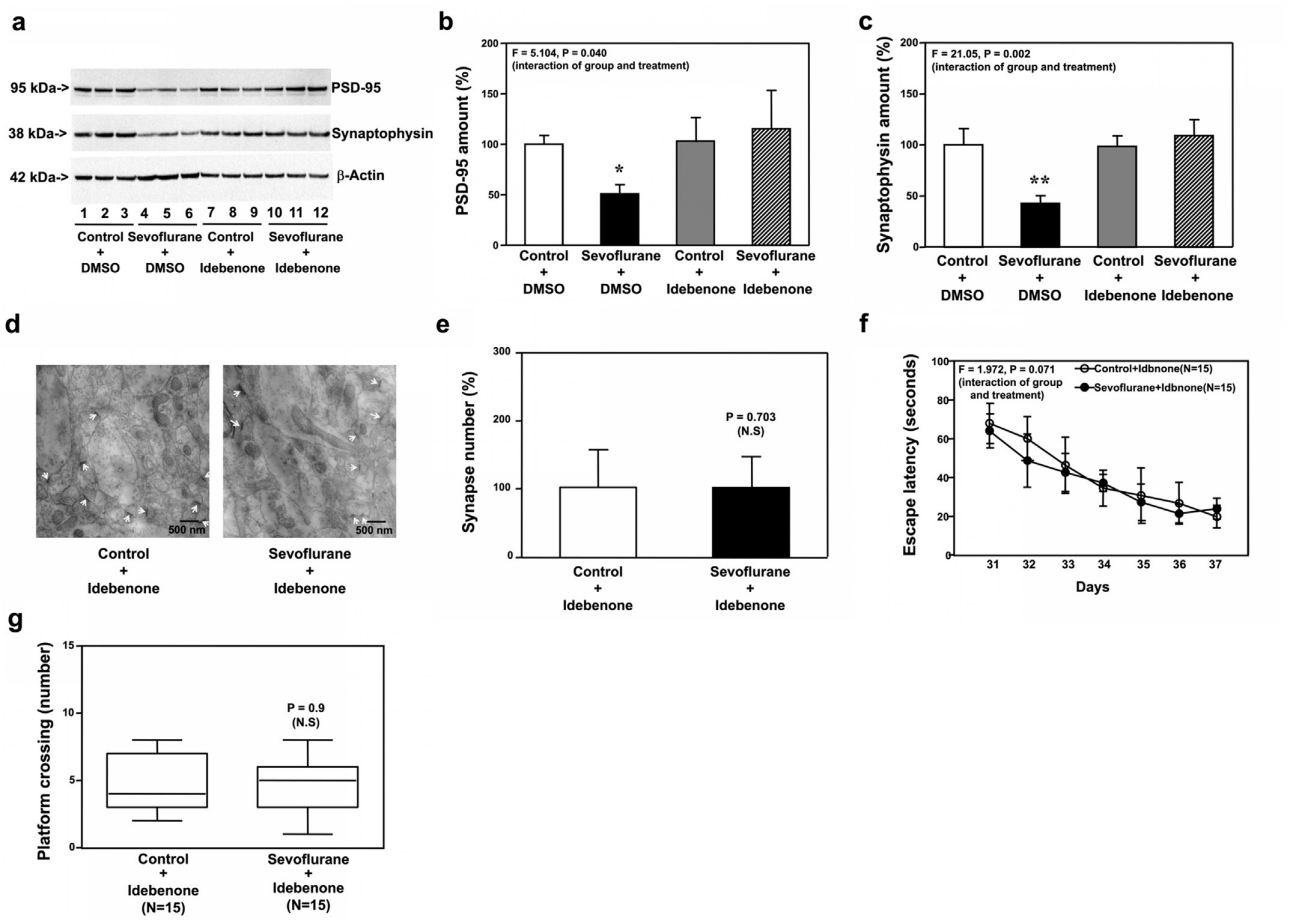
**Effects of idebenone on sevoflurane-induced changes in the P30 WT mice**. **a.** Sevoflurane (lanes 4 to 6) decreased the protein amounts of PSD-95 and synaptophysin as compared to the control condition (lanes 1 to 3) in the hippocampi of WT mice harvested at P30. In the WT mice pretreated with idebenone, however, sevoflurane (lanes 10 to 12) did not significantly change the protein amounts of PSD-95 and synaptophysin as compared to the control condition (lanes 7 to 9) in the hippocampi of the WT mice pretreated with idebenone harvested at P30. Quantification of the Western blot showed that sevoflurane (black bar) decreased the protein amounts of PSD-95 (**b**) and synaptophysin (**c**) as compared to the control condition (white bar) in the hippocampi of WT mice harvested at P30. In the WT mice pretreated with idebenone, however, sevoflurane (stripe bar) did not apparently change the amounts of PSD-95 (**b**) and synaptophysin (**c**) as compared to the control condition (gray bar) in the hippocampi of the WT mice pretreated with idebenone harvested at P30. **d**. Sevoflurane did not decrease the number of synapses as compared to the control condition in the hippocampi of the WT mice pretreated with idebenone harvested at P30. **e.** Quantification of the electron microscope ultrastructure images showed that sevoflurane (black bar) did not significantly change the number of synapses as compared to the control condition (white bar) in WT mice pretreated with idebenone. **f.** The effects of sevoflurane on the escape latency of the WT mice pretreated with idebenone in MWM from P31 to P37. **g.** The effects of sevoflurane on the platform crossing number of the WT mice pretreated with idebenone in MWM on P38. Postsynaptic density 95, PSD-95; Morris Water Maze, MWM; Wild type, WT. N ​= ​9 in each group of the biochemistry studies and N ​= ​15 in each group of the behavioral studies.

Similarly, idebonone mitigated the sevoflurane-induced reduction in amounts of PSD-95 (Fig. 10a and b, P ​= ​0.040, N ​= ​9) and synaptophysin (Fig. 10a and c, P ​= ​0.002, N ​= ​9) in hippocampi of WT mice at P30. Sevoflurane did not reduce synapse number in hippocampi of WT mice pretreated with idebenone at P30 (Fig. 10d and e) and did not induce cognitive impairment in WT mice pretreated with idebenone from P31 to P37 (Fig. 10f and g).

## 4 Discussion

**Fig. 11:**
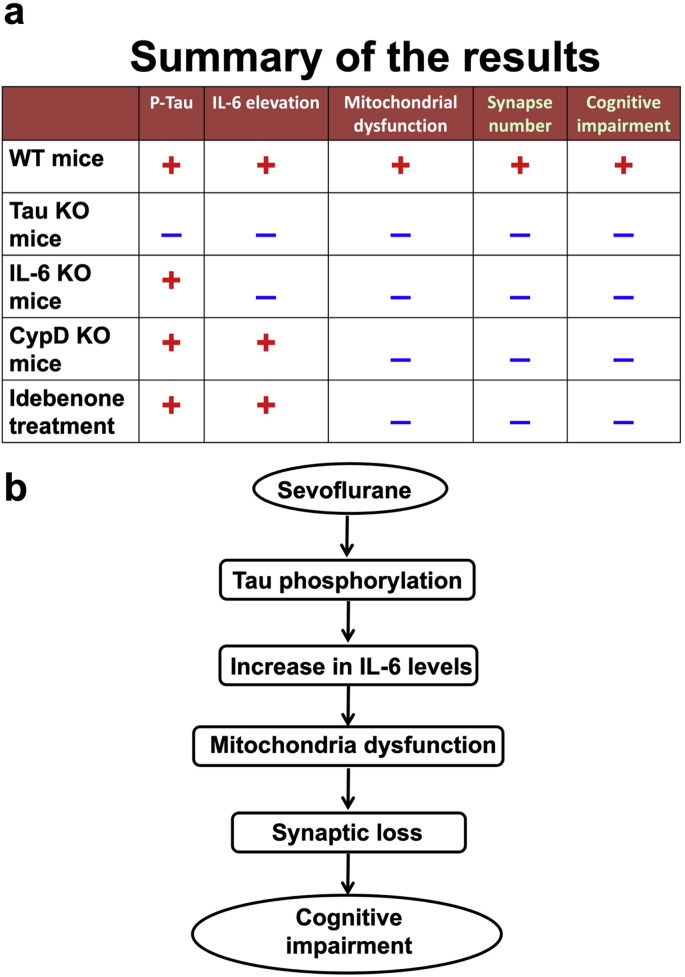
**Hypothesized pathway**. **a.** Sevoflurane increased Tau phosphorylation and elevation of IL-6 amounts, induced mitochondrial dysfunction, reduction in synapse number and cognitive impairment in the WT young mice. Sevoflurane caused none of these changes in the Tau KO mice. In the IL-6 KO mice, however, sevoflurane still increased Tau phosphorylation, but did not induce the mitochondrial dysfunction, reduction in synapse number and cognitive impairment. Finally, in the CypD KO mice and the WT mice pretreated with idebenone, sevoflurane increased Tau phosphorylation and elevation of IL-6 amounts, but did not induce mitochondrial dysfunction, reduction in synapse number and cognitive impairment. **b**. These data suggest that sevoflurane in the young mice first induces Tau phosphorylation, which then causes elevation of IL-6 amounts, leading to mitochondrial dysfunction. The mitochondrial dysfunction then produces a reduction in the number of synapse and cognitive impairment in the young mice.

Employing sevoflurane as a clinically relevant tool, and WT, Tau KO, IL-6 KO, and CypD KO young mice as the approaches, we demonstrated the interactions of Tau phosphorylation, IL-6 accumulation and mitochondrial dysfunction and the effects of such interactions on synapse number and cognitive impairment in young mice. The findings in the present studies suggest that Tau phosphorylation causes IL-6 elevation, which induces mitochondrial dysfunction, leading to synaptic loss and cognitive impairment in the young mice following sevoflurane anesthesia (Fig. 11). Moreover, idebenone mitigated the sevoflurane-induced mitochondrial dysfunction, synaptic loss, and cognitive impairment, but not the sevoflurane-induced increase in Tau phosphorylation and IL-6 elevation. These results with idebenone could be particularly important because the specific inhibition of Tau phosphorylation is not available and general anti-inflammation treatment (e.g., non-steroidal anti-inflammatory drugs) may not be practical owing to the potential side effects, e.g., deficiency of wound healing and bleeding. These findings suggest that idebenone could be used to treat or prevent the cognitive impairment in young mice and merits further investigation.

First, we found that sevoflurane increased amounts of phosphorylated Tau (pTau) (Tau-PS202/PT205) and IL-6, and induced mitochondrial dysfunction, synaptic loss and cognitive impairment in young WT mice (Figs. 1 and 2). In the Tau KO mice, however, sevoflurane caused a lesser degree of these effects (Figs. 3 and 4). In the IL-6 KO mice, sevoflurane caused a lesser degree of mitochondrial dysfunction, synaptic loss, and cognitive impairment, but still increased Tau phosphorylation in hippocampi of the mice (Figs. 5 and 6). Moreover, sevoflurane increased Tau phosphorylation and IL-6, but did not cause mitochondrial dysfunction, synaptic loss and cognitive impairment in CypD KO mice (Figs. 7 and 8). These data illustrated that Tau phosphorylation could induce cognitive impairment via IL-6 elevation, mitochondrial dysfunction, and synaptic loss in young mice.

Furthermore, sevoflurane-induced IL-6 elevation was dependent, at least partially, on Tau, but independent of mitochondrial dysfunction. Sevoflurane-induced mitochondrial dysfunction was dependent, at least partially, on Tau protein and IL-6 increase, but independent of synaptic loss. Together, these data demonstrated the interactions of Tau phosphorylation, IL-6 elevation and mitochondrial dysfunction and such interactions could lead to reduction in synapse number and cognitive impairment in young mice. These results revealed one of the underlying mechanisms by which Tau phosphorylation caused cognitive impairment in young mice.

Anesthesia and Tau phosphorylation, inflammation and mitochondrial dysfunction in the brain tissues of young mice have been extensively studied (Vutskits and Xie, 2016). Previous studies have shown that sevoflurane can increase Tau phosphorylation (Hu et al., 2013; Huang et al., 2018; Tao et al., 2014; Yang et al., 2020a, 2020b; Yu et al., 2009, Yu et al., 2020) and cause IL-6 elevation (Shen et al., 2013; Yang et al., 2020a, 2020b; Zhang et al., 2013; Zheng et al., 2013) in brain tissues of young mice. Consistently, the present study also showed that sevoflurane increased the amounts of Tau-PS202/PT205 and IL-6. Moreover, the present study used Tau KO and IL-6 KO mice to demonstrate that the sevoflurane-induced Tau phosphorylation was not dependent on IL-6; but the sevoflurane-induced elevation of IL-6 was dependent on Tau protein.

Anesthesia with isoflurane, nitrous oxide, and midazolam in P7 rats was able to induce an enlargement of mitochondria, impairment of the structural integrity, increases in their complex IV activity, and decreases in the regional distribution in presynaptic neuronal profiles (Sanchez et al., 2011). Other studies also showed that the same anesthesia could increase ROS amounts and mitochondrial fission, leading to disturbance in mitochondrial morphogenesis (Boscolo et al., 2013). The anesthesia with 2.5% sevoflurane in P7, P10 and P13 rats caused a reduction in the mitochondrial density in the brain tissues of the rats (Boscolo et al., 2013). Finally, the multiple exposures to sevoflurane in young mice reduced ATP concentrations in the brain tissues of the young mice (Xu et al., 2017). The findings from these studies were consistent with the results of the current study that sevoflurane induced mitochondrial dysfunction in brain tissues of the young mice. However, the present study further demonstrated that sevoflurane-induced mitochondrial dysfunction was partially dependent on Tau and IL-6. Notably, there could be many cell deaths from P6 to P8 in the brain tissues of mice (Bandeira et al., 2009; Mosley et al., 2017). Our recent study demonstrated that knockout of CypD attenuated the sevoflurane-induced cell death and cognitive impairment in young mice (Zhang et al., 2019). The future studies should include the investigation of whether the mitochondrial dysfunction and overexpression of CypD can further promote Tau phosphorylation and IL-6 elevation in the brain tissues of young mice, forming a vicious cycle of Tau phosphorylation, IL-6 elevation and mitochondrial dysfunction in young mice.

The underlying mechanism by which Tau phosphorylation leads to elevation of IL-6 remains largely to be determined. A recent study demonstrated that microglia can uptake, process, and release Tau (Hopp et al., 2018). Thus, it is possible that Tau phosphorylation can cause more microglia activation, leading to elevation of IL-6. Future studies to test this hypothesis are warranted.

Several studies showed that general anesthesia was able to cause loss of both excitatory and inhibitory synapse in brains of young mice (between P5 and P10) (Briner et al., 2011; Head et al., 2009; Lunardi et al., 2010). Moreover, these changes in synapse lasted for a long time and were associated with impaired transmission in the neuronal networks (Amrock et al., 2015; DiGruccio et al., 2015; Jevtovic-Todorovic et al., 2003; Lunardi et al., 2010). Consistently, the data from the current studies showed that sevoflurane in young mice caused a synaptic loss in the brain tissues of the mice. However, the results from the current studies further demonstrated for the first time that sevoflurane-induced synaptic loss was dependent, at least partially, on Tau, IL-6, and mitochondrial dysfunction in the brain tissues of the young mice.

Since sevoflurane increased Tau phosphorylation and IL-6, and induced mitochondrial dysfunction, which then led to synaptic loss and cognitive impairment in the young mice, we could target Tau phosphorylation, IL-6 elevation, and mitochondria to prevent or treat the cognitive impairment in young mice. However, specific inhibition of Tau phosphorylation is not available. Furthermore, non-specific anti-inflammatory agents have potential side effects.

Idebenone is a synthetic analog of co-enzyme Q10 (Rauchova et al., 2008). It has been reported that idebenone can protect mitochondrial dysfunction [(Erb et al., 2012; Orsucci et al., 2011), reviewed in (Pfeffer et al., 2013)]. Consistently, we showed that idebenone mitigated the sevoflurane-induced mitochondrial dysfunction, synaptic loss, and cognitive impairment, without altering Tau phosphorylation and IL-6 elevation, in the present studies (Figs. 9 and 10). Clinical investigation showed that idebenone could treat dementia and memory impairment in patients without dementia (Voronkova and Meleshkov, 2009). Thus, translationally, the data from the current studies suggest the potential use of idebenone in the treatment and/or prevention of the cognitive impairment in young brain, pending further investigation. Future studies to determine whether drugs that enhance mitochondrial function can prevent or treat the cognitive impairment in children are warranted.

The study has several limitations. First, Tau protein can be highly phosphorylated at many serine or threonine sites in developmental brain (Yu et al., 2009, Yu et al., 2020). In the present study, we only assessed the effects of sevoflurane on the amounts of Tau-PS202/PT205 but not other sites of Tau phosphorylation. However, the objective of the current studies was not fully characterizing the effects of sevoflurane on Tau phosphorylation but rather using the Tau-PS202/PT205, as a representative, to determine the interactions of Tau phosphorylation, IL-6 elevation, and mitochondrial dysfunction, and to assess whether such interactions can cause synaptic loss and cognitive impairment in young mice. The future studies will include assessing the interactions of other sites of pTau with IL-6 elevation and mitochondrial dysfunction. Second, we did not determine the total Tau amounts following sevoflurane because our previous studies had illustrated that the same sevoflurane treatment increased the amounts of Tau-PS202/PT205 without significantly altering the amounts of total Tau (Tao et al., 2014).

In conclusion, using Tau KO, IL-6 KO, and CypD KO young mice as the transgenic approaches and employing anesthetic sevoflurane as a clinically relevant tool, we demonstrated that Tau phosphorylation might cause IL-6 elevation, which induced mitochondrial dysfunction, leading to synaptic loss and cognitive impairment in young mice. Moreover, we showed that idebenone mitigated the sevoflurane-induced mitochondrial dysfunction, synaptic loss, and the cognitive impairment in the mice. These findings would promote further research to determine the underlying mechanisms by which Tau phosphorylation induced cognitive impairment, as well as the targeted interventions in young brain.

## Funding

This research was supported by the 10.13039/100000002National Institutes of Health (grant number HD086977 to Zhongcong Xie) and was performed in the Department of Anesthesia, Critical Care and Pain Medicine at 10.13039/100005294Massachusetts General Hospital and 10.13039/100006691Harvard Medical School. Dr. Jie Zhang was partially supported by the 10.13039/501100001809National Natural Science Foundation of China (grant number 81500931) for her efforts in writing and submitting the manuscript. The funders had no role in the study design; data collection and analyses; the writing or editing of the manuscript; or in the decision to publish.

## Author contributions

Literature search and Study design: JZ and ZX; Methodology (including data collection, analysis and interpretation): JZ, YD, LH, XX; Figures: JZ and YD; Writing-Original Draft: JZ, ZX; Writing-Review & Editing: FL, YZ, SGS; Resources: ZX; Supervision: YZ, ZX. JZ and YD have contributed equally to the study.

## Declaration of competing interest

The authors declared no conflict of interests related to the studies. Dr. Zhongcong Xie is a consultant for Baxter, Novartis, Shanghai Jiaotong University and Tongji University.

## References

[bib1] Ali S.A., Aly H.F., Faddah L.M., Zaidi Z.F. (2012). Dietary supplementation of some antioxidants against hypoxia. World J. Gastroenterol..

[bib2] Amrock L.G., Starner M.L., Murphy K.L., Baxter M.G. (2015). Long-term effects of single or multiple neonatal sevoflurane exposures on rat hippocampal ultrastructure. Anesthesiology.

[bib3] Bandeira F., Lent R., Herculano-Houzel S. (2009). Changing numbers of neuronal and non-neuronal cells underlie postnatal brain growth in the rat. Proc. Natl. Acad. Sci. U. S. A..

[bib4] Basu R., Taylor M.R., Williams M.E. (2015). The classic cadherins in synaptic specificity. Cell Adhes. Migrat..

[bib5] Boscolo A., Milanovic D., Starr J.A., Sanchez V., Oklopcic A., Moy L., Ori C.C., Erisir A., Jevtovic-Todorovic V. (2013). Early exposure to general anesthesia disturbs mitochondrial fission and fusion in the developing rat brain. Anesthesiology.

[bib6] Boscolo A., Starr J.A., Sanchez V., Lunardi N., DiGruccio M.R., Ori C., Erisir A., Trimmer P., Bennett J., Jevtovic-Todorovic V. (2012). The abolishment of anesthesia-induced cognitive impairment by timely protection of mitochondria in the developing rat brain: the importance of free oxygen radicals and mitochondrial integrity. Neurobiol. Dis..

[bib7] Briner A., Nikonenko I., De Roo M., Dayer A., Muller D., Vutskits L. (2011). Developmental Stage-dependent persistent impact of propofol anesthesia on dendritic spines in the rat medial prefrontal cortex. Anesthesiology.

[bib8] Brookes P.S., Yoon Y., Robotham J.L., Anders M.W., Sheu S.S. (2004). Calcium, ATP, and ROS: a mitochondrial love-hate triangle. Am. J. Physiol. Cell Physiol..

[bib9] Cen C., Luo L.D., Li W.Q., Li G., Tian N.X., Zheng G., Yin D.M., Zou Y., Wang Y. (2018). PKD1 promotes functional synapse formation coordinated with N-cadherin in Hippocampus. J. Neurosci..

[bib10] Chaturvedi R.K., Flint Beal M. (2013). Mitochondrial diseases of the brain. Free Radic. Biol. Med..

[bib11] Chazeau A., Garcia M., Czondor K., Perrais D., Tessier B., Giannone G., Thoumine O. (2015). Mechanical coupling between transsynaptic N-cadherin adhesions and actin flow stabilizes dendritic spines. Mol. Biol. Cell.

[bib12] Chen X., Levy J.M., Hou A., Winters C., Azzam R., Sousa A.A., Leapman R.D., Nicoll R.A., Reese T.S. (2015). PSD-95 family MAGUKs are essential for anchoring AMPA and NMDA receptor complexes at the postsynaptic density. Proc. Natl. Acad. Sci. U. S. A..

[bib13] Clare R., King V.G., Wirenfeldt M., Vinters H.V. (2010). Synapse loss in dementias. J. Neurosci. Res..

[bib14] Coley A.A., Gao W.J. (2018). PSD95: a synaptic protein implicated in schizophrenia or autism?. Prog. Neuro-Psychopharmacol. Biol. Psychiatry.

[bib15] DiGruccio M.R., Joksimovic S., Joksovic P.M., Lunardi N., Salajegheh R., Jevtovic-Todorovic V., Beenhakker M.P., Goodkin H.P., Todorovic S.M. (2015). Hyperexcitability of rat thalamocortical networks after exposure to general anesthesia during brain development. J. Neurosci..

[bib16] Dong Y., Zhang G., Zhang B., Moir R.D., Xia W., Marcantonio E.R., Culley D.J., Crosby G., Tanzi R.E., Xie Z. (2009). The common inhalational anesthetic sevoflurane induces apoptosis and increases beta-amyloid protein levels. Arch. Neurol..

[bib17] Du H., Guo L., Fang F., Chen D., Sosunov A.A., McKhann G.M., Yan Y., Wang C., Zhang H., Molkentin J.D., Gunn-Moore F.J., Vonsattel J.P., Arancio O., Chen J.X., Yan S.D. (2008). Cyclophilin D deficiency attenuates mitochondrial and neuronal perturbation and ameliorates learning and memory in Alzheimer’s disease. Nat. Med..

[bib18] Du H., Guo L., Zhang W., Rydzewska M., Yan S. (2011). Cyclophilin D deficiency improves mitochondrial function and learning/memory in aging Alzheimer disease mouse model. Neurobiol. Aging.

[bib19] Erb M., Hoffmann-Enger B., Deppe H., Soeberdt M., Haefeli R.H., Rummey C., Feurer A., Gueven N. (2012). Features of idebenone and related short-chain quinones that rescue ATP levels under conditions of impaired mitochondrial complex I. PloS One.

[bib20] Fischer G., Wittmann-Liebold B., Lang K., Kiefhaber T., Schmid F.X. (1989). Cyclophilin and peptidyl-prolyl cis-trans isomerase are probably identical proteins. Nature.

[bib21] Gainutdinov T., Molkentin J.D., Siemen D., Ziemer M., Debska-Vielhaber G., Vielhaber S., Gizatullina Z., Orynbayeva Z., Gellerich F.N. (2015). Knockout of cyclophilin D in Ppif(-)/(-) mice increases stability of brain mitochondria against Ca(2)(+) stress. Arch. Biochem. Biophys..

[bib22] Haefeli R.H., Erb M., Gemperli A.C., Robay D., Courdier Fruh I., Anklin C., Dallmann R., Gueven N. (2011). NQO1-dependent redox cycling of idebenone: effects on cellular redox potential and energy levels. PloS One.

[bib23] Hao Y., Shabanpoor A., Metz G.A. (2017). Stress and corticosterone alter synaptic plasticity in a rat model of Parkinson’s disease. Neurosci. Lett..

[bib24] Head B.P., Patel H.H., Niesman I.R., Drummond J.C., Roth D.M., Patel P.M. (2009). Inhibition of p75 neurotrophin receptor attenuates isoflurane-mediated neuronal apoptosis in the neonatal central nervous system. Anesthesiology.

[bib25] Hong S., Beja-Glasser V.F., Nfonoyim B.M., Frouin A., Li S., Ramakrishnan S., Merry K.M., Shi Q., Rosenthal A., Barres B.A., Lemere C.A., Selkoe D.J., Stevens B. (2016). Complement and microglia mediate early synapse loss in Alzheimer mouse models. Science.

[bib26] Hopp S.C., Lin Y., Oakley D., Roe A.D., DeVos S.L., Hanlon D., Hyman B.T. (2018). The role of microglia in processing and spreading of bioactive tau seeds in Alzheimer’s disease. J. Neuroinflammation.

[bib27] Hu Z.Y., Jin H.Y., Xu L.L., Zhu Z.R., Jiang Y.L., Seal R. (2013). Effects of sevoflurane on the expression of tau protein mRNA and Ser396/404 site in the hippocampus of developing rat brain. Paediatr. Anaesth..

[bib28] Huang C., Chu J.M., Liu Y., Chang R.C., Wong G.T. (2018). Varenicline reduces DNA damage, tau mislocalization and post surgical cognitive impairment in aged mice. Neuropharmacology.

[bib29] Janz R., Sudhof T.C., Hammer R.E., Unni V., Siegelbaum S.A., Bolshakov V.Y. (1999). Essential roles in synaptic plasticity for synaptogyrin I and synaptophysin I. Neuron.

[bib30] Jevtovic-Todorovic V., Hartman R.E., Izumi Y., Benshoff N.D., Dikranian K., Zorumski C.F., Olney J.W., Wozniak D.F. (2003). Early exposure to common anesthetic agents causes widespread neurodegeneration in the developing rat brain and persistent learning deficits. J. Neurosci..

[bib31] Johri A., Beal M.F. (2012). Mitochondrial dysfunction in neurodegenerative diseases. J. Pharmacol. Exp. Therapeut..

[bib32] Kaufman A.C., Salazar S.V., Haas L.T., Yang J., Kostylev M.A., Jeng A.T., Robinson S.A., Gunther E.C., van Dyck C.H., Nygaard H.B., Strittmatter S.M. (2015). Fyn inhibition rescues established memory and synapse loss in Alzheimer mice. Ann. Neurol..

[bib33] Kovalenko M., Milnerwood A., Giordano J., St Claire J., Guide J.R., Stromberg M., Gillis T., Sapp E., DiFiglia M., MacDonald M.E., Carroll J.B., Lee J.M., Tappan S., Raymond L., Wheeler V.C. (2018). HttQ111/+ huntington’s disease knock-in mice exhibit brain region-specific morphological changes and synaptic dysfunction. J Huntingtons Dis.

[bib34] Lu H., Liufu N., Dong Y., Xu G., Zhang Y., Shu L., Soriano S.G., Zheng H., Yu B., Xie Z. (2017). Sevoflurane acts on ubiquitination-proteasome pathway to reduce postsynaptic density 95 protein levels in young mice. Anesthesiology.

[bib35] Lunardi N., Ori C., Erisir A., Jevtovic-Todorovic V. (2010). General anesthesia causes long-lasting disturbances in the ultrastructural properties of developing synapses in young rats. Neurotox. Res..

[bib36] Mosley M., Shah C., Morse K.A., Miloro S.A., Holmes M.M., Ahern T.H., Forger N.G. (2017). Patterns of cell death in the perinatal mouse forebrain. J. Comp. Neurol..

[bib37] Murmu R.P., Li W., Szepesi Z., Li J.Y. (2015). Altered sensory experience exacerbates stable dendritic spine and synapse loss in a mouse model of Huntington’s disease. J. Neurosci..

[bib38] Nagaoka A., Suno M., Shibota M., Kakihana M. (1989). Effects of idebenone on neurological deficits, local cerebral blood flow, and energy metabolism in rats with experimental cerebral ischemia. Arch. Gerontol. Geriatr..

[bib39] Nagaoka T., Ohashi R., Inutsuka A., Sakai S., Fujisawa N., Yokoyama M., Huang Y.H., Igarashi M., Kishi M. (2014). The Wnt/planar cell polarity pathway component Vangl2 induces synapse formation through direct control of N-cadherin. Cell Rep..

[bib40] Orsucci D., Mancuso M., Ienco E.C., LoGerfo A., Siciliano G. (2011). Targeting mitochondrial dysfunction and neurodegeneration by means of coenzyme Q10 and its analogues. Curr. Med. Chem..

[bib41] Pfeffer G., Horvath R., Klopstock T., Mootha V.K., Suomalainen A., Koene S., Hirano M., Zeviani M., Bindoff L.A., Yu-Wai-Man P., Hanna M., Carelli V., McFarland R., Majamaa K., Turnbull D.M., Smeitink J., Chinnery P.F. (2013). New treatments for mitochondrial disease-no time to drop our standards. Nat. Rev. Neurol..

[bib42] Rauchova H., Drahota Z., Bergamini C., Fato R., Lenaz G. (2008). Modification of respiratory-chain enzyme activities in brown adipose tissue mitochondria by idebenone (hydroxydecyl-ubiquinone). J. Bioenerg. Biomembr..

[bib43] Sanchez V., Feinstein S.D., Lunardi N., Joksovic P.M., Boscolo A., Todorovic S.M., Jevtovic-Todorovic V. (2011). General anesthesia causes long-term impairment of mitochondrial morphogenesis and synaptic transmission in developing rat brain. Anesthesiology.

[bib44] Shen X., Dong Y., Xu Z., Wang H., Miao C., Soriano S.G., Sun D., Baxter M.G., Zhang Y., Xie Z. (2013). Selective anesthesia-induced neuroinflammation in developing mouse brain and cognitive impairment. Anesthesiology.

[bib45] Sun L.S., Li G., Miller T.L., Salorio C., Byrne M.W., Bellinger D.C., Ing C., Park R., Radcliffe J., Hays S.R., DiMaggio C.J., Cooper T.J., Rauh V., Maxwell L.G., Youn A., McGowan F.X. (2016). Association between a single general anesthesia exposure before age 36 Months and neurocognitive outcomes in later childhood. J. Am. Med. Assoc..

[bib46] Tao G., Zhang J., Zhang L., Dong Y., Yu B., Crosby G., Culley D.J., Zhang Y., Xie Z. (2014). Sevoflurane induces tau phosphorylation and glycogen synthase kinase 3beta activation in young mice. Anesthesiology.

[bib47] Valero T. (2014). Mitochondrial biogenesis: pharmacological approaches. Curr. Pharmaceut. Des..

[bib48] Voronkova K.V., Meleshkov M.N. (2009). Use of Noben (idebenone) in the treatment of dementia and memory impairments without dementia. Neurosci. Behav. Physiol..

[bib49] Vutskits L., Xie Z. (2016). Lasting impact of general anaesthesia on the brain: mechanisms and relevance. Nat. Rev. Neurosci..

[bib50] Xiang Q., Li X.H., Fang X.X., Jia J., Ren J., Dong Y.C., Ou-Yang C. (2018). Alterations of synaptic plasticity in aged rats: evidence of functional and morphological studies. Technol. Health Care.

[bib51] Xu G., Lu H., Dong Y., Shapoval D., Soriano S.G., Liu X., Zhang Y., Xie Z. (2017). Coenzyme Q10 reduces sevoflurane-induced cognitive deficiency in young mice. Br. J. Anaesth..

[bib52] Yang M., Lian N., Yu Y., Wang Y., Xie K., Yu Y. (2020). Coenzyme Q10 alleviates sevofluraneinduced neuroinflammation by regulating the levels of apolipoprotein E and phosphorylated tau protein in mouse hippocampal neurons. Mol. Med. Rep..

[bib53] Yang M., Tan H., Zhang K., Lian N., Yu Y., Yu Y. (2020). Protective effects of Coenzyme Q10 against sevoflurane-induced cognitive impairment through regulating apolipoprotein E and phosphorylated Tau expression in young mice. Int. J. Dev. Neurosci..

[bib54] Yu Y., Run X., Liang Z., Li Y., Liu F., Liu Y., Iqbal K., Grundke-Iqbal I., Gong C.X. (2009). Developmental regulation of tau phosphorylation, tau kinases, and tau phosphatases. J. Neurochem..

[bib55] Yu Y., Yang Y., Tan H., Boukhali M., Khatri A., Yu Y., Hua F., Liu L., Li M., Yang G., Dong Y., Zhang Y., Haas W., Xie Z. (2020). Tau contributes to sevoflurane-induced neurocognitive impairment in neonatal mice. Anesthesiology.

[bib58] Zhang L., Zhang J., Yang L., Dong Y., Zhang Y., Xie Z. (2013). Isoflurane and sevoflurane increase interleukin-6 levels through the nuclear factor-kappa B pathway in neuroglioma cells. Br. J. Anaesth..

[bib57] Zhang J., Dong Y., Zhou C., Zhang Y., Xie Z. (2015). Anesthetic sevoflurane reduces levels of hippocalcin and postsynaptic density protein 95. Mol. Neurobiol..

[bib59] Zhang Y., Lu P., Liang F., Liufu N., Dong Y., Zheng J.C., Xie Z. (2019). Cyclophilin D contributes to anesthesia neurotoxicity in the developing brain. Front Cell Dev Biol.

[bib60] Zhang Y., Xu Z., Wang H., Dong Y., Shi H.N., Culley D.J., Crosby G., Marcantonio E.R., Tanzi R.E., Xie Z. (2012). Anesthetics isoflurane and desflurane differently affect mitochondrial function, learning, and memory. Ann. Neurol..

[bib61] Zheng H., Dong Y., Xu Z., Crosby G., Culley D.J., Zhang Y., Xie Z. (2013). Sevoflurane anesthesia in pregnant mice induces neurotoxicity in fetal and offspring mice. Anesthesiology.

[bib62] Zhu X., Perry G., Smith M.A., Wang X. (2013). Abnormal mitochondrial dynamics in the pathogenesis of Alzheimer’s disease. J Alzheimers Dis.

